# *yggS* Encoding Pyridoxal 5′-Phosphate Binding Protein Is Required for *Acidovorax citrulli* Virulence

**DOI:** 10.3389/fmicb.2021.783862

**Published:** 2022-01-11

**Authors:** Yuanjie Wang, Yuqiang Zhao, Liming Xia, Lin Chen, Yajie Liao, Baohui Chen, Yiyang Liu, Weirong Gong, Yanli Tian, Baishi Hu

**Affiliations:** ^1^College of Plant Protection and Key Laboratory of Integrated Management of Crop Diseases and Pests, Ministry of Education, Nanjing Agricultural University, Nanjing, China; ^2^Institute of Botany, Jiangsu Province and Chinese Academy of Sciences (Nanjing Botanical Garden Mem. Sun Yat-sen), Nanjing, China; ^3^Plant Protection and Quarantine Station of Province, Nanjing, China

**Keywords:** *Aicdovorax citrulli*, virulence, YggS, type III secretion (T3S), biological processes

## Abstract

Bacterial fruit blotch, caused by seed-borne pathogen *Acidovorax citrulli*, poses a serious threat to the production of cucurbits globally. Although the disease can cause substantial economic losses, limited information is available about the molecular mechanisms of virulence. This study identified that, a random transposon insertion mutant impaired in the ability to elicit a hypersensitive response on tobacco. The disrupted gene in this mutant was determined to be *Aave_0638*, which is predicted to encode a YggS family pyridoxal phosphate-dependent enzyme. YggS is a highly conserved protein among multiple organisms, and is responsible for maintaining the homeostasis of pyridoxal 5′-phosphate and amino acids in cells. *yggS* deletion mutant of *A. citrulli* strain XjL12 displayed attenuated virulence, delayed hypersensitive response, less tolerance to H_2_O_2_ and pyridoxine, increased sensitivity to antibiotic β-chloro-D-alanine, and reduced swimming. In addition, RNA-Seq analysis demonstrated that *yggS* was involved in regulating the expression of certain pathogenicity-associated genes related to secretion, motility, quorum sensing and oxidative stress response. Importantly, YggS significantly affected type III secretion system and its effectors *in vitro*. Collectively, our results suggest that YggS is indispensable for *A.citrulli* virulence and expands the role of YggS in the biological processes.

## Introduction

Bacterial fruit blotch (BFB), a seed-borne disease caused by *Acidovorax citrulli*, has caused substantial economic losses to the global cucurbit industry (especially melon and watermelon) (Schaad et al., 1978, 2008; Willems et al., 1992). Since the first report in the United States (Webb and Goth, 1965), BFB has spread worldwide mainly through the international movement of contaminated seeds, which are the main inoculum sources for BFB outbreak (Hopkins and Thompson, 2002; Burdman and Walcott, 2012). However, to date, effective management strategies including watermelon cultivars with significant level resistance to BFB are not commercially available (Bahar et al., 2009a; Burdman and Walcott, 2012; Ge et al., 2021). Despite the fact that BFB is a serious threat to cucurbit crops production, the mechanism of pathogenicity of *A. citrulli* is largely unknown. Therefore, it is critical to elucidate the pathogenicity factors of *A. citrulli* at the molecular level in order to develop effective BFB management strategies.

The availability of a complete genome sequence of *A. citrulli* strain AAC00-1 (GenBank NC_008752) greatly enhances the investigation of pathogenesis. So far, several diverse virulence-related factors have been characterized for this phytobacterium. Protein secretion systems and motility mediated by pili and flagella are indispensable for phytobacterial pathogenicity (Mattick, 2002; Chaban et al., 2015; Pfeilmeier et al., 2016) and *A. citrulli* is no exception. Previous studies have confirmed that disruption of the T3SS abolished the pathogenicity of *A. citrulli* (Ren et al., 2009; Johnson et al., 2011; Liu et al., 2012; Zhang et al., 2018). Type III-secreted effectors (T3Es) delivered into host cells *via* T3SS promote the invasion of pathogens through interference of the cell metabolisms and/or suppression of the host immune responses (Feng and Zhou, 2012; Macho and Zipfel, 2015). The annotation of genome of AAC00-1 indicates that there are at least 11 T3Es genes in this phytobacterium (Eckshtain-Levi et al., 2014). Recently, the discovery of a wide arsenal of T3Es placed *A. citrulli* among the “richest” bacteria in terms of T3E cargo (Jiménez-Guerrero et al., 2020). More than 50 T3Es sharing similarity with known T3Es from other pathogenic bacteria were revealed in *A. citrulli* strain M6 by computational approach, and seven new putative T3Es were further validated as real effectors through T3SS-dependent translocation assay (Jiménez-Guerrero et al., 2020). Two effectors, AopN and AopP, were confirmed to inhibit plant immunity by interacting with ClHIPP, CILTP and ClWRKY6 in watermelon, respectively (Zhang et al., 2020a,b). In addition, type II and VI secretion systems were reported to play key roles in *A. citrulli* virulence (Johnson et al., 2009; Tian et al., 2015). Polar flagellum and type IV pili (TFP) of *A. citrulli* are required for motility, colonization ability and virulence, and the lack of ability to synthesize TFP causes phenotypic variation in *A. citrulli* (Bahar et al., 2009b,^2011^; Rosenberg et al., 2018). In addition to the above-mentioned classical apparatus related to bacterial pathogenicity, other factors such as ferric uptake regulator (FurA) (Liu et al., 2019), quorum sensing (QS) (Wang et al., 2016), and bifunctional chorismate mutase/prephenate dehydratase (Cmp) (Kim et al., 2020), have been reported to contribute to *A. citrulli* virulence. These advances have improved our understanding of the complex pathogenic mechanisms.

YggS is a member of a conserved COG0325 protein family of PLP (pyridoxal 5′-phosphate)-binding proteins and widely present and highly conversed in various organisms (Ito et al., 2013; Darin et al., 2016; Labella et al., 2017). Although this protein family is similar to bacterial alanine racemase and eukaryotic ornithine decarboxylase in structure, no enzymatic activity was detected (Eswaramoorthy et al., 2003; Ito et al., 2013; Tremino et al., 2017). Several studies have shown that YggS is responsible for maintaining the homeostasis of PLP, a biologically active form of vitamin B_6_ and an essential cofactor in various kinds of enzymes (Prunetti et al., 2016; Ito et al., 2019). The lack of YggS or its orthologs in this protein family exhibit pleiotropic phenotypes in multiple organisms by unknown mechanisms. In *E. coli*, the absence of *yggS* leads to perturbations in levels of amino acid metabolic and α-ketobutyrate (Ito et al., 2013, 2016, 2019), the accumulation of the PLP precursor pyridoxine 5′-phosphate (PNP) and the sensitivity to pyridoxine (PN), which can be suppressed by pyridoxal (PL) (Prunetti et al., 2016). PipY, a homolog of YggS, was studied in *Synechococcus elongates*, and the *pipY* mutant was more susceptible to PN, but also to the antibiotics D-cycloserine (DCS) and β-chloro-D-alanine (BCDA), both targeting key PLP-holoenzymes. The addition of D-alanine or L-alanine was shown to rescue the susceptibility to both antibiotics (Labella et al., 2017; Tremino et al., 2017). Recently, a study on *Salmonella enterica* lacking YggS suggested that other than the accumulation of endogenous PNP, approximately 10-fold more PLP were detected in growth medium as compared to the wild-type strain (Vu et al., 2020). In addition, in humans, vitamin B_6_-dependent epilepsy is attributed to the variation in *PLBP* (formerly called *PROSC*), a homolog of *yggS*. This mutant alters the level of vitamin B6 and neurotransmitters (Darin et al., 2016; Johnstone et al., 2019). Despite the important biological function of YggS in organisms, the role of YggS associated with biochemistry or virulence has not been investigated in *A. citrulli*.

The current study was initiated to identify novel factors related to *A. citrulli* virulence. By screening transposon (Tn5)-insertion library, we obtained a mutant strain of *A. citrulli* with an altered HR phenotype in tobacco and reduced virulence to melon compared to the wild-type strain. The gene disrupted by transposon insertion mutation was identified as *Aave_0638* that encoded a YggS family pyridoxal phosphate-dependent enzyme (YggS). *Aave_0638* was homologous with YggS in *E. coli* by BLASTp analysis in National Center for Biotechnology Information (NCBI). The data indicates that YggS was involved in oxidative stress response, motility, the sensitivity to PN and BCDA and the regulation of T3SS in *A. citrulli*. The RNA-Seq revealed that the absence of YggS had a greater impact on T3SS and its effectors *in vitro*. In the current study, we uncovered the first insights into the role of *yggS* in virulence of *A. citrulli*.

## Materials and Methods

### Bacterial Strains, Culture Conditions, and Plant Material

Bacterial strains and plasmids used in this study are listed in Supplementary Table 1. Bacteria were cultured in Luria-Bertani (LB) agar or broth medium (Sambrook et al., 1989). *Acidovorax citrulli* and *Escherichia coli* were cultured at 28 and 37°C, respectively. When required, the appropriate dose of antibiotics were added to media at the following final concentrations: rifamycin (Rif) 100 μg/mL, kanamycin (Km) 50 μg/mL, gentamicin (Gm) 50 μg/mL, chloramphenicol (Cm) 20 μg/mL and ampicillin (Amp), 100 μg/mL. The turbidity of the cell suspensions was measured by optical density at 600 nm using a spectrophotometer (Thermo Scientific, Waltham, MA, United States). For pathogenicity assays, melon (cv. Huanghou) seeds were planted in soil mixed with 50% vermiculite and grown under standard greenhouse conditions including 24°C and 12 h of natural light until inoculated. The inoculated seedlings were incubated in growth chamber with 28°C, 85% relative humidity (RH) and 12 h of fluorescent light.

### Construction of a Transposon-Insertion Library of *A. citrulli* XjL12 and Identification of Disrupted Genes

To obtain a high efficiency, random insertion mutant library, triparental mating was conducted. In brief, cells of wild-type *A. citrulli* strain XjL12, and *E. coli* containing pUTKm and pRK600, respectively, were mixed and cultured on nitrocellulose membrane (NCM) on an LB agar plate. After 48 h, the lawn was harvested, washed and plated on LB agar supplemented with Rif and Km. The resulting mutants were confirmed by *A. citrulli*-specific primers WFB1/WFB2 (Walcott and Gitaitis, 2000) and KMF/KMR for the Tn5 transposon. Mutants were grown overnight and bacterial cell suspensions were adjusted to OD_600_ = 0.3 (3 × 10^8^ CFU/mL). Approximately 10 μL of each cell suspension was infiltrated into tobacco leaves to test for HR induction. The inoculated tobacco leaves were grown at 28°C and observed per 8 h.

To identify the disrupted gene of the Tn5 insertion mutant of *A. citrulli*, a plasmid rescue method was employed as described previously (Bahar et al., 2009b) with some modifications. Briefly, the genomic DNA of the *A. citrulli* mutant was extracted and digested with *Pst*I restriction enzyme that is unable to digest the Tn5 region, but is able to excise the genome into fragments. The digestion products were ligated into pUC19 digested with the same restriction enzyme, and the recombinant vector was introduced into DH5α. Due to the existence of a kanamycin (KM) cassette in Tn5, the flanking regions of Tn5 were identified by backward sequencing using the specific primers Tn5-F/Tn5-R for KM cassette. The sequence was then blast searched against the *A. citrulli* AAC00-1 genome in NCBI using Blastn. All primer sequences used in this study are listed in Supplementary Table 2.

### Construction of a Δ*yggS* Mutant and Complemented Strains of *Acidovorax citrulli*

The Δ*yggS* mutant of *A. citrulli* was generated through homologous recombination as described previously (Johnson et al., 2011). The *yggS* gene (locus tag: *Aave_0638*) is located in region 688,056 to 688,781 in the *A. citrulli* AAC00-1 genome (GenBank NC_008752). The upstream and downstream fragments of *yggS* were amplified using primer pairs (*yggS*-upF/*yggS*-upR, *yggS*-downF/*yggS*-downR) designed using Primer3 online^1^, and then digested with restriction enzymes (*Hin*dIII and *Xba*I, *Bam*HI and *Kpn*I, respectively). The Km fragment was cloned from pET30 using Km cassette primer pair Km-F/Km-R and digested with *Xba*I and *Bam*HI simultaneously. Three fragments were ligated with suicide vector pEX18 (digested with *Hin*dIII and *Kpn*I), and the recombinant vector pEX18*yggS*Km was introduced into *E. coli* BW20676 for biparental mating with wild-type XjL12. The resulting *A. citrulli* mutant Δ*yggS* was confirmed by PCR assay using primers *yggS*-upF/*yggS*-downR.

To construct complemented strains, the *yggS* expression vector was first constructed using pBBR1-MCS-5. The *yggS* promoter was predicted using the online promoter prediction program^2^. The sequence containing the promoter and open reading frame of *yggS*, was generated using primers *yggS*-F/*yggS*-R and digested with *Kpn*I and *BamH*I and then ligated with pBBR-MCS-5 digested with the same enzymes. Afterwards, the recombinant vector pBBR-*yggS* verified by Sanger sequencing was introduced into *E. coli* BW20676 for biparental mating with the *A. citrulli* mutant Δ*yggS*. All primer sequences used in this assay are listed in Supplementary Table 2.

### *In vitro* Bacterial Growth Assays

*Acidovorax citrulli* growth was measured in LB broth. After culturing overnight, the strains were harvested and adjusted to an OD_600_ of 0.1 with sterilized water. The bacterial suspensions were added to fresh LB broth at 1:100 (vol/vol) and then incubated at 28°C with shaking at 220 rpm. A growth curve was investigated by measuring bacterial cell turbidity using a spectrophotometer (BioPhotometer, Eppendorf) at OD_600_ at 2 h intervals until the cultures reached the plateau phase. In this assay, three biological replicates were performed and the experiment was repeated three times independently.

### Biofilm Formation

The ability of biofilm formation of *A. citrulli* was measured as described previously (Bahar et al., 2009b). Briefly, all strains cultured overnight were harvested and washed twice with sterilized water. Forty microliters of each bacterial suspension at OD_600_ of 1.0 were added to 4 mL LB broth, in 12-well polyvinyl chloride (PVC) plates and incubated at 28°C. After 48 h, the cell suspension in each well was removed and the plate was dried at 80°C for 20 min. The biofilms attached to the plate well walls were stained with 1% methyl violet for 50 min and then solubilized in absolute ethyl alcohol. The biofilms were quantified by measuring solutions at OD_590_ with a Microplate Reader (Synergy H1, Biotek). Three replicates for each strain were performed per experiment and the experiment was repeated three times.

### Bacterial Motility Assay

Swimming assay was conducted as a previous protocol (Wang et al., 2016) with some modifications. For the swimming assay, 5 μL of cell suspensions at OD_600_ of 0.3 (3 × 10^8^ CFU/mL) were deposited in the center of oligotrophic medium containing 0.3% agar and incubated at 28°C. The diameters of three colonies of each strain were measured after 48 h. Each strain was tested three times per experiment and this experiment was conducted three times.

### Hypersensitive Response Assay

To determine the ability of *A. citrulli* to induce hypersensitive response (HR), cell suspensions were injected into the leaves of *Nicotiana tabacum* as described previously (Liu et al., 2019). In brief, *A. citrulli* strains grown in LB overnight were washed with sterilized water and adjusted to OD_600_ = 0.3. About 10 μL of cell suspensions were syringe-infiltrated into the leaves of *N*. *tabacum* grown at 28°C and HR was observed after 12 h. Three leaves at the same leaf position on the different stem were inoculated by tested strains. The experiment was conducted three times.

### H_2_O_2_, Antibiotic and Pyridoxine (PN) Susceptibility Assays

To evaluate *A. citrulli* sensitivity to some chemical compounds, the inhibition zone method was used. For sensitivity to H_2_O_2_ and antibiotics, 1 mL of cell suspensions at OD_600_ of 0.3 were added to 50 mL LB agar medium and poured into sterilized petri dishes. A sterilized paper disk, approximately 4 mm in diameter, was placed in the center of each plate containing bacteria. Ten microliters of H_2_O_2_ (5% and 10%) or antibiotics were dropped on the disk. After incubating at 28°C for 48 h, the diameters of inhibition zones were measured. Similarly, sensitivity to 1 M PN was tested as described above except for the concentration of cell suspension. To make the inhibition zone more easily visible, cell suspension added to LB agar medium was at an OD_600_ = 0.1. In this assay, the final concentrations of tested antibiotics were as follows (antibiotic/concentration in mg/mL): ampicillin/10, β-chloro-D-alanine (BCDA)/100, chloramphenicol/4, D-cycloserine (DCS)/10, gentamicin/12.5, spectinomycin/50, tetracycline/5. Each strain was tested three times per experiment and the experiment was conducted three times.

### Virulence Assays

In order to evaluate the effect of *yggS* on *A. citrulli* virulence, three inoculation methods were performed as described previously (Liu et al., 2019).

i. Cotyledon infiltration assay: Each *A. citrulli* suspension (approximately 1 × 10^4^ CFU/mL) was injected into cotyledons of five one-week-old melon (cv. Huanghou) seedlings and the plants were incubated in a growth chamber at 28°C, 85% RH and exposed to 12 h of fluorescent light daily. BFB symptoms were observed at 3, 5, and 7 days postinoculation (dpi).
ii. Seed-to-seedling transmission assay: Germinating melon (cv. Huanghou) seeds were immersed in 2 mL of each cell suspension diluted to 1 × 10^8^ CFU/mL until the seeds were air-dried at room temperature. Twenty seeds inoculated with each strain were planted in one cup and incubated in growth chamber with 28°C, 85% RH and 12 h of fluorescent light. After one week, seedlings were visually observed.
iii. Seedling spray inoculation: When the second euphylla of melon (cv. Huanghou) seedlings fully emerged (about 3 weeks old), seedlings were inoculated with cell suspension at OD_600_ of 0.3 by spraying. About 50 mL cell suspension of each strain was spray-inoculated evenly onto twenty seedlings per experiment. Seedlings were incubated at 100% RH for two days and then at 85% RH. After one week, the euphylla were observed for BFB symptoms. Each seedling was evaluated for BFB severity based on disease index (DI) as described previously (Araújo et al., 2005), with modifications. Briefly, disease severity scale ranged from 0 to 5: 0 for no symptoms; 1, 2, 3, 4 for necrotic lesions on approximately 25, 50, 75, 100% of the leaves, respectively; 5 for complete death of seedling. The DI was calculated based on the formula: DI = Σ(A × B) × 100/ΣB × 5 (where A: disease class (0, 1, 2, 3, 4, 5); B: the number of seedlings in the corresponding disease class). This experiment was conducted three times.

### Bacterial Colonization of Melon Cotyledons and Seeds Assay

A previous established protocol (Tian et al., 2015) was used to assess the role of *yggS* in *A. citrulli* colonization of melon seedlings with slight modification. For cotyledon colonization assay, cell suspensions of each *A. citrulli* strain (approximately 1 × 10^3^ CFU/mL) were injected into at least twenty-five cotyledons of melon (cv. Huanghou) seedlings per experiment. Five 5-mm disks were collected from cotyledons injected with each strain at 0, 1, 2, 3, 4 dpi and triturated in 1 mL of buffer in the sterilized 1.5-mL centrifuge tubes. Homogenate was 10-fold serially diluted with sterile water and 100 μL of homogenate was spread on LB plates with appropriate antibiotics. Resulting *A. citrulli* colonies were counted after two days. For seed colonization assays, the seeds (cv. Huanghou) in the assay were disinfected with 5% H_2_O_2_ for 20 min before germination to prevent microbe contamination. The front end of the germinating seeds were opened gently and five microliters of cell suspensions (approximately 1 × 10^5^ CFU/mL) were inoculated into the melon seeds. Each strain was inoculated into at least thirty-five melon seeds per experiment. The inoculated seeds were placed on moist filter paper. Five seeds inoculated with each strain were collected at 0, 1, 2, 3, 4, 5, 6, 7 dpi, and each seed was shaken for 10 min in a sterilized 2-mL centrifuge tube containing 1 mL sterilized water. Seed homogenate was 10-fold serially diluted with sterilized water and 100 μL was spread on LB plates with appropriate antibiotics.

Bacterial colonization was quantified by calculating the area under population dynamics curve (AUPDC) as follows: AUPDC = $\sum_{i=1}^{n}\left[\left(Y_{i}+Y_{i+1}\right)/2\right]\times \left(X_{i+1}-X_{i}\right)$ (Bjarko and Line, 1988). (*X_i_*: the value of horizontal coordinates at *i*th observation; *Y*_*i*_: the value of vertical coordinates at the *i*th observation; n: the total number of observation). This assay was repeated three times.

### Transcriptome Sequencing and Data Analysis

To determine the regulatory mechanism of YggS in *A. citrulli*, RNA-Seq was conducted commercially by Beijing Allwegene Technology Company Limited (Beijing, China). Briefly, total RNA were extracted from *A. citrulli* strains that were cultured in LB broth to OD_600_ = 1.0 using the TRIzol method (TIANGEN BIOTECH, Bejing). RNA was quantified by Agilent 2100 (Agilent Technologies, CA, USA), and the quality and integrity were detected by NanoDrop spectrophotometer (IMPLEN, CA, United States). Ribosomal RNA (rRNA) was removed from qualified RNA sample using Vazyme Ribo-off rRNA depletion kit (Bacteria) (Vazyme biotech, United States). Subsequently, the sequencing libraries were generated using NEBNext ULtraTM RNA library Prep Kit (NEB, United States). Library quality was assessed on the Agilent Bioanalyzer 2100 system. The qualified library was sequenced by Illumina Hiseq 4000 platform. The reads cleaned by Trimmomatic V0.33 were mapped to *A. citrulli* AAC00-1 genome (GenBank NC_008752) by Bowtie2 V2.2.6. Resulting data were subjected to DESeq R package (1.10.1) for analyzing differential expression genes between mutant and wild-type strain. Genes with an adjusted *P*-value < 0.05 found by DESeq were assigned as differentially expressed. Gene Ontology (GO) enrichment analysis and Kyoto Encyclopedia of Genes and Genomes (KEGG) enrichment analysis were performed by GOSeq and KOBAS, respectively. GO term and KEGG pathway with corrected *P* value < 0.05 were defined as significantly enriched by the DEGs. Each strain was analyzed in three biological repetitions.

### Quantitative Real-Time PCR Analysis

To validate the result of RNA-Seq, quantitative real-time PCR analysis (qPCR) was conducted. The culture condition for bacteria growth was the same as described in the transcriptome sequencing and data analysis. Total *A. citrulli* RNA was extracted from bacteria using the bacterial RNA kit (OMEGA), and the concentration of RNA was measured by spectrophotometer (Nanodrop One, Thermo Scientific). cDNA was synthesized and purified using HiScript III RT SuperMix kit (Vazyme, Nanjing, China) and diluted to 100 ng/μL for qPCR with ChamQ Universal SYBR qPCR Master Mix (Vazyme, Nanjing, China). The qPCR assay was carried out in a real-time PCR machine (7,500, Applied Biosystems) as the following program: 95°C for 30 s (1 cycle); 95°C for 10 s, 60°C for 30 s (40 cycles); melting curve profiled from 60°C to 95°C to check the specialty of reaction. The primers of selected genes used in the assay are listed in Supplementary Table 2. 16s ribosomal RNA gene was used as a reference gene. Each sample was tested four times per experiment and experiments were conducted three times independently. Relative gene expression was calculated in the method of 2^−ΔΔct^ as described previously (Livak and Schmittgen, 2001).

### Statistical Analyses

All data were analyzed by SPSS Statistics 26. The one-way analysis of variance (ANOVA) and least Significant Difference (LSD) test were used to determine the significant difference in disease index, biofilm assay, motility assay and AUPDC. Differences with *p* < 0.05 were considered significant.

## Results

### YggS Is Conserved and Clusters Strongly With PilT in Multiple Organisms

**FIGURE 1:**
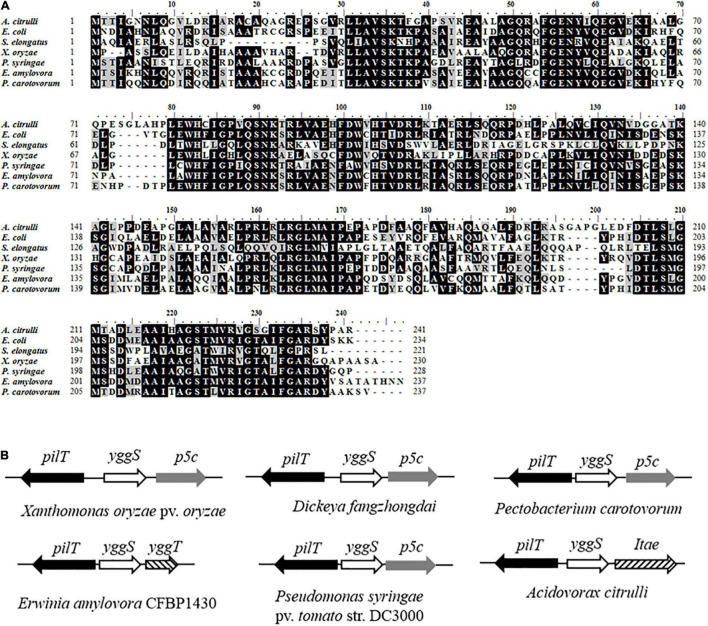
Amino acid sequences of YggS and physical clustering of *yggS* genes. **(A)** Multiple alignments of YggS amino acid sequences for *Acidovorax citrulli* and other important bacteria including *Escherichia coli* str. K-12 substr. MG1655, *Synechococcus elongates* PCC7942, *Pseudomonas syringae* pv. *tomato* str. DC3000, *Xanthomonas oryzae* pv. *oryzae* PXO99A, *Dickeya fangzhongdai* strain DSM 101947, *Erwinia amylovora* CFBP1430 and *Pectobacterium carotovorum* PC1. The analysis was performed by BioEdite V7.0.5. Among these sequences, amino acids marked with black are conserved, and marked with gray are highly homologous. **(B)** Physical clustering of *yggS* genes in phytopathogenic bacteria. The direction of the arrow is consistent with the direction of the gene transcription.

Through library screening, an *A. citrulli* mutant named sk24 that was unable to induce a HR on tobacco at 12 h after inoculation was obtained (Supplementary Figure 1). By subcloning and sequence analysis, we identified the gene disrupted by transposon as *Aave_0638* (Supplementary File 1). This gene putatively encodes YggS family pyridoxal phosphate-dependent enzyme, which exhibits structual similarity to the *N*-terminal domain of alanine racemase (EC 5.1.1.1). Multiple sequence alignment shows that the amino acid sequence of YggS from *A. citrulli* has high identity among the tested bacteria including *E. coli* (Figure 1A). By BLASTP sequence homology analysis, YggS displays 53.94, 38.97, 49.57, 53.78, 55.46, and 55.14% sequence identity with homologs from *E. coil* MG1655, *S. elongates* PCC7942, *X. oryzae* PXO99A, *P. syringae* DC3000, *E. amylovora* CFBP1430 and *P. carotovorum* PC1, respectively. Furthermore, previous studies have shown that *yggS* clusters strongly with genes such as those related to metabolism, including cell division and cell wall (Prunetti et al., 2016). We observed the position of *yggS* on genome and found that *yggS* and *pilT*, encoding ATPase-mediating pilus retraction and disassembly, are clustered together. This genetic organization appears to be conserved for serval phytopathogenic bacteria (Figure 1B).

### *yggS* Contributes to *Acidovorax citrulli* Virulence

**FIGURE 2:**
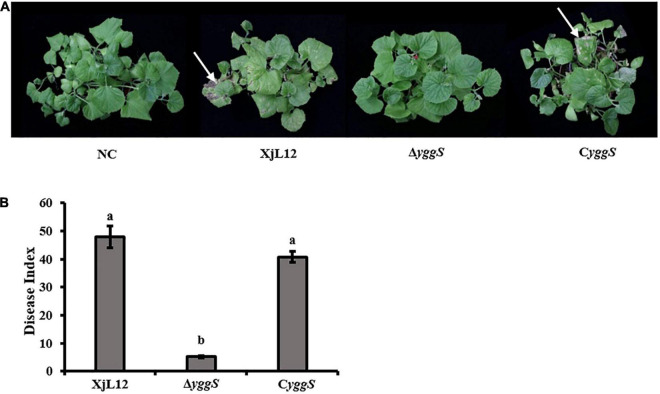
Spray inoculation assay. **(A)** *A. citrulli* suspension (∼1 × 10^8^ CFU/mL) were inoculated onto the euphylla of melon by spray inoculation. The phenotypes were photographed after the melon seedlings were allowed to grow for 7 days after inoculation. The red arrows indicate the BFB symptoms. **(B)** Disease index for each treatment is represented by a histogram. The bars represent means and lines indicate standard errors of the mean. XjL12: wild-type strain, Δ*yggS*: *yggS* deletion mutant; C*yggS*: complemented strain of Δ*yggS*. Letters above each bar represent significant differences as determined by the LSD test (*P* < 0.05).

**FIGURE 3:**
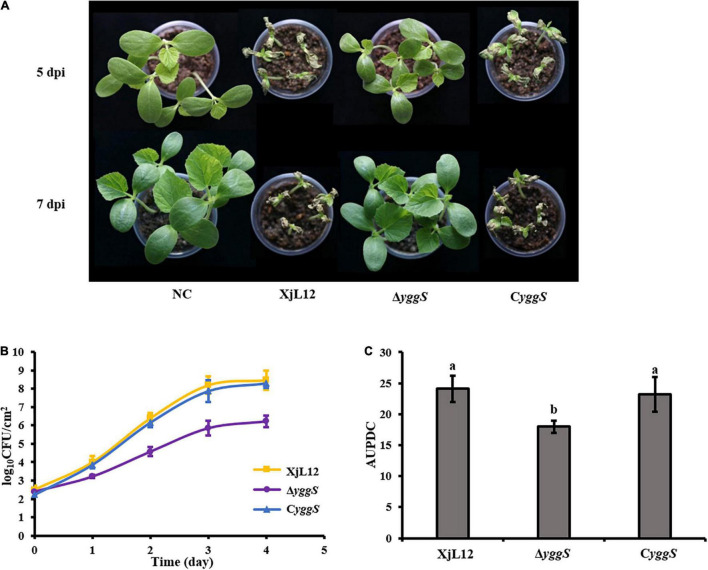
Effect of *yggS* on the ability of *A. citrulli* to colonize in melon cotyledons. **(A)** *A. citrulli* cell suspensions (∼1 × 10^4^ CFU/mL) were injected into the cotyledons of melon seedlings. The images represent seedling symptoms at 5 and 7 days post-inoculation (dpi). **(B)** The melon cotyledons were injected with bacterial suspensions (∼1 × 10^5^ CFU/mL) and the bacterial populations were quantified for five days. **(C)** Histogram of area under population dynamics curve (AUPDC) calculated for *A. citrulli* colonization of cotyledons. In the graph, the data points represent means and the tines represent the standard errors of the mean. In the histogram, the bars represent means and lines indicate the standard errors of the mean. XjL12: wild-type strain, Δ*yggS*: *yggS* deletion mutant; C*yggS*: complemented strain of Δ*yggS*. Letters above the bar represent significant differences as determined by the LSD test (*P* < 0.05).

To investigate the effect of *yggS* on *A. citrulli* virulence, we compared the pathogenicity of the wild-type strain XjL12, the mutant strain Δ*yggS* and complemented strain C*yggS* by conducting cotyledon injection and spray inoculation of melon seedling euphylla. The results of the spray inoculation assay showed that mutation of *yggS* impaired the virulence of *A.citrulli* (Figure 2A). Seven days after inoculation, necrotic lesions developed on the euphylla sprayed with *A. cirulli* strains XjL12 and C*yggS*, while the euphylla sprayed with Δ*yggS* showed mild necrotic symptoms. The disease indices (DI) caused by XjL12, Δ*yggs* and C*yggS* were 47.87, 5.15, and 40.78, respectively. The DI of Δ*yggS* was significantly lower than the wild-type and complemented strains (*P* < 0.05) (Figure 2B). As shown in Figure 3A, the cotyledons injected with Δ*yggS* showed no symptoms, similar to the negative control (NC) at 5 days after inoculation. Meanwhile, melon cotyledons injected with XjL12 and C*yggS* showed typical BFB symptoms, including seedling blight/collapse. By 7 dpi, there were no visible BFB symptoms on melon cotyledons injected with the mutant strain. In agreement with the results of cotyledon injection, Δ*yggS* impaired its ability to colonize melon cotyledons. The bacterial populations of XjL12, Δ*yggS* and C*yggS* were approximately 2.94 × 10^8^, 1.69 × 10^6^, 1.95 × 10^8^ CFU/cm^2^ by 4 dpi, respectively (Figure 3B). As expected, AUPDC data showed that the population of Δ*yggS* was significantly less than that of XjL12 and C*yggS* in cotyledons (*p* < 0.05) (Figure 3C).

### *yggS* Is Required for Seed-to-Seedling Transmission of BFB

**FIGURE 4:**
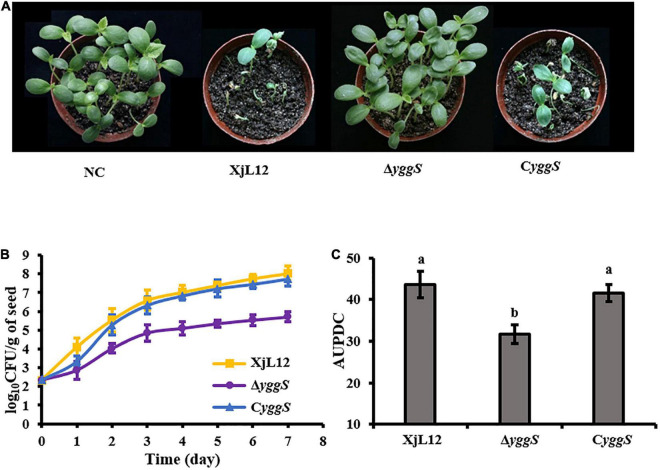
Role of *yggS* in seed-to-seedling transmission BFB in melon and in colonization in melon seeds by *A. citrulli*. **(A)** Effect of *yggS* on seed-to-seeding transmission of *A. citrulli* on melon. Melon seeds (n = 20) were innoculated by soaking in bacterial cell suspensions (∼1 × 10^8^ CFU/mL) and then planted. One week after planting, BFB symptoms were observed and photographed. **(B)** Germinating melon seeds were inoculated with approximately 5 uL of bacterial suspension (∼1 × 10^5^ CFU/mL) and the bacterial populations in seeds were quantified each day for seven days. Each strain was used to inoculate five seeds and each experiment was repeated three times. **(C)** Histogram of AUPDC calculated bacterial colonization of melon seed. In the graph, the data points represent means and the tines represent the standard errors of the mean. In the histogram, the bars represent means and lines indicate the standard errors of the mean. XjL12: wild-type strain, Δ*yggS*: *yggS* deletion mutant; C*yggS*: complemented strain of Δ*yggS*. Letters above the bars represent significant differences as determined by the LSD test (*P* < 0.05).

In the seed-to-seedling transmission assay, melon seeds infiltrated with *A. cirtulli* strains were planted at 20 seeds per pot and the percentage of dead seedlings was calculated one week after planting. Most seedlings from seeds inoculated with wild-type strain XjL12 and complemented strain C*yggS* were died, while all the seedlings inoculated with Δ*yggS* survived without visible BFB symptoms (Figure 4A). The reduced seed-to-seedling transmission indicated that it is likely that the loss of *yggS* impaired the colonization ability in melon seeds. So, we assayed the population dynamics of *A. citrulli* in germinating seeds and the bacterial population in inoculated seeds were quantified per 24 h after inoculation (Figure 4B). The average populations of XjL12, Δ*yggS* and C*yggS* were approximately 1.07 × 10^8^, 5.12 × 10^5^, 5.37 × 10^7^ CFU/g of seed by one week after inoculation, respectively. AUPDC data showed that the populations of Δ*yggS* was significantly less than XjL12 and C*yggS* in seeds (*p* < 0.05) (Figure 4C). These results suggest that *yggS* plays an important role in seed-to-seedling transmission of BFB and seed colonization by *A. citrulli*.

### The Absence of *yggS* Causes Delay in *Acidovorax citrulli* Growth, Which Can Be Restored by Exogenous PL

**FIGURE 5:**
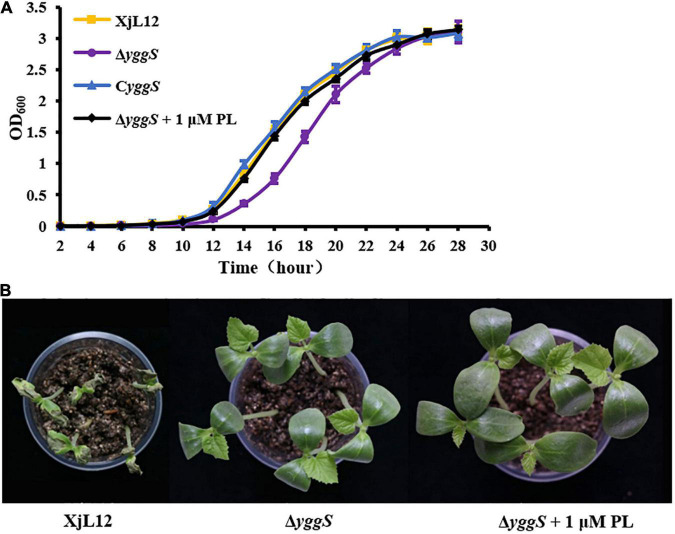
Effect of pyridoxal (PL) on Δ*yggS* growth in LB and virulence on melon seedlings. **(A)** Assay of *A. citrulli* growth rate. All strains were cultured overnight, diluted to OD_600_ = 0.1 with sterilized water, and then transferred to fresh LB at a ratio of 1:100. Optical density of cell suspensions was measured at 2 h intervals. The data points represent means and the tines represent the standard errors of the mean for three experiments. Each experiment was repeated three times. **(B)** Effect of *yggS* on virulence of *A.citrulli* on melon cotyledons through exogenous addition of PL. Δ*yggS* suspension (∼1 × 10^5^ CFU/mL) mixed 1 μM PL was injected into cotyledons of seedlings, and XjL12 and Δ*yggS* were used as controls. The images represent the seedlings at 5 days post-inoculation. XjL12: wild-type strain, Δ*yggS*: *yggS* deletion mutant; C*yggS*: complemented strain of Δ*yggS*.

For most organism but *Pseudomonas aeruginosa*, YggS is dispensable for growth (Rusmini et al., 2014; Vu et al., 2020). To investigate the role of YggS in *A. citrulli* growth, the ability of the *A. citrulli* strains Δ*yggS*, wild-type XjL12 and C*yggS* complement to grow in LB medium was compared by measuring the optical density of cell suspensions. Δ*yggS* reached the exponential growth phase after 14 h and reached the stationary phase at 26 h. On the other hand, the wild-type and complemented strains reached the exponential and stationary phases approximately at 12 h and 24 h, respectively (Figure 5A). The date indicated that *yggS* also plays a dispensable role for *A. citrulli* despite of the slight reduction in growth of Δ*yggS*. In addition, Δ*yggS* cultured in the LB broth amended with 1 μM PL returned to wild-type growth (Figure 5A), while PL was unable to enhance the growth of wild-type or complemented strain (Supplementary Figure 2).

To determine if PL could restore compromised virulence, Δ*yggS* was syringe-inoculated into melon cotyledons along with 1 μM PL. Although PL restored growth of Δ*yggS*, it did not restore pathogenicity on melon cotyledons (Figure 5B). This result showed that the effect of *yggS* on bacterial growth may not be related to virulence attenuation.

### The Absence of *yggS* Reduces Swimming Motility in *Acidovorax* c*itrulli*

**FIGURE 6:**
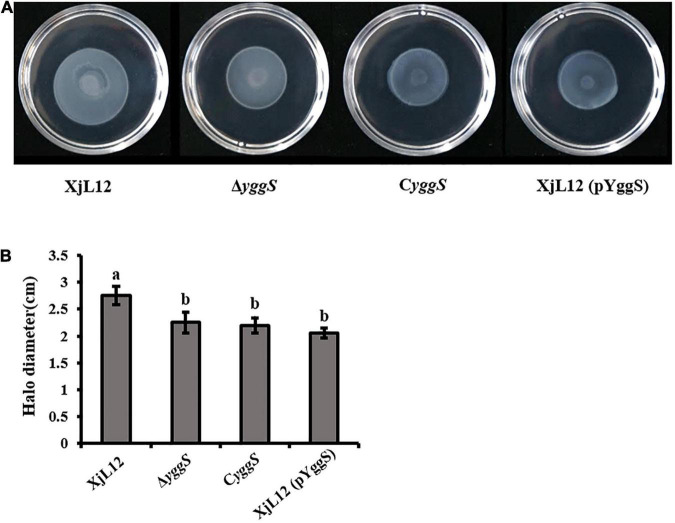
Effect of *yggS* on *Acidovorax citrulli* motility. **(A)** Swimming motility of *A. citrulli* strains. Bacterial suspensions diluted to 3 × 10^8^ CFU/mL with sterilized water were deposited 5 μL of suspensions in the center of the 0.3% agar plate. The swimming colonies were photographed after 48 h. **(B)** The swimming colonies diameter of *A. citrulli* strains were measured after 48 h. The vertical bars in the histogram represent standard errors of the mean for three experiments. Each experiment was repeated three times. XjL12: wild-type strain, Δ*yggS*: *yggS* deletion mutant; C*yggS*: complemented strain of Δ*yggS*. XjL12 (pYggS): *yggS*-overexpressed strain. Letters above the bars represent significant differences as determined by the LSD test (*P* < 0.05).

Previous studies have confirmed that bacterial motility plays a key role in pathogenicity of *A. citrulli* (Bahar et al., 2009b; Bahar et al., 2011). *A. citrulli* strains produced near circular colonies *via* swimming motility using the soft agar plate assay (Figure 6A). The diameters of XjL12, Δ*yggS*, C*yggS*, and XjL12 (pYggS) were 2.75 ± 0.17, 2.25 ± 0.19, 2.20 ± 0.14, and 2.05 ± 0.09 cm, respectively (Figure 6B). The diameters of the swimming motility colony produced by Δ*yggS* and overexpressed strain XjL12 (pYggS) were significantly smaller than the colony produced by wild-type strain (*p* < 0.05) (Figure 6B). However, the complemented strain with pBBRMCS-5, containing the *yggS* gene, failed to restore the swimming motility phenotype.

### Δ*yggS* Is More Susceptible to Pyridoxine (PN), H_2_O_2_ and BCDA

**FIGURE 7:**
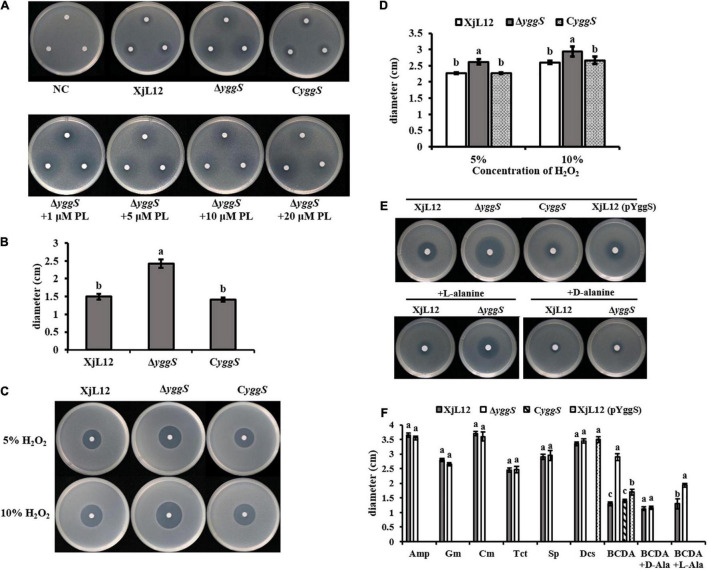
The role of *yggS* in the sensitivity of *Acidovorax citrulli* to selected stressors. **(A)** The toxicity of pyridoxine (PN) (in the absence or presence of pyridoxal (PL)) to *A. citrulli* strains. **(B)** The diameter of inhibition zones caused by PN. All phenotypes were observed after incubation for 48 h. **(C)** Sensitivity of *A. citrulli* strains to H_2_O_2_. Two different concentrations of H_2_O_2_ were dropped in the center of the plate containing *A. citrulli* strains. **(D)** H_2_O_2_ sensitivity was measured by the diameter of the zone of bacterial growth inhibition. **(E)** Representative images illustrating the sensitivity of *A. citrulli* strains to BCDA (in the absence or presence of L-alanine or D-alanine). **(F)** Diameters of *A. citrulli* inhibition zones for each antibiotic were represented by histogram. The bars in the histogram represent mean inhibition zone and lines represent standard errors of the mean of three experiments. XjL12: wild-type strain, Δ*yggS*: *yggS* deletion mutant; C*yggS*: complemented strain of Δ*yggS*. XjL12 (pYggS): *yggS*-overexpressed strain. Letters above the bars represent significant differences as determined by the LSD test (*P* < 0.05). Amp = ampicillin, BCDA = β-chloro-D-alanine, Cm = chloramphenicol, DCS = D-cycloserine, Gm = gentamicin, Sp = spectinomycin, Tct = tetracycline.

In *E. coli*, excess PN led to a zone of inhibition with Δ*yggS*, but not with the wild-type strain. We observed a similar phenomenon with *A. citrulli* (Figure 7A). The mean diameter of the inhibition zones produced by Δ*yggS* (2.42 ± 0.12 cm) was greater (*p* < 0.05) than the diameters of the wild type XjL12 (1.50 ± 0.08 cm) and the complementation strain C*yggS* (1.41 ± 0.06 cm) (Figure 7B). This result suggested that the deletion of *yggS* increases the sensitivity to PN. However, the sensitivity to PN could not be rescued by exogenous addition of PL, and even with a range of PL concentrations; this was different to the observations reported for *E. coli* (Figure 7A; Prunetti et al., 2016). To determine if YggS is involved in oxidative stress tolerance in *A. citrulli*, we measured the sensitivity to H_2_O_2_ (Figure 7C). Compared to the wild-type and complemented strains, the diameters of inhibition zone for the mutant were greater (*p* < 0.05) at 5 and 10% H_2_O_2_; conversely, the diameter of C*yggS* was not significantly different to that of XjL12 (Figure 7D). The results suggest that deletion of *yggS* increases sensitivity to H_2_O_2_ and that *yggS* contributes to oxidative stress tolerance of *A.citrulli*. In order to determine whether *yggS* inactivation affects the sensitivity of *A. citrulli* to antibiotics, we tested 7 common antibiotics, including D-cycloserine (DCS) and β-chloro-D-alanine (BCDA) targeting alanine racemase. As shown in Figures 7E,F, the *yggS* mutant was more sensitive than XjL12 and C*yggS* to BCDA, but not to the other antibiotics (Supplementary Figure 3). Further, the sensitivity of mutant to BCDA was reduced by exogenous addition of L-alanine or D-alanine (Figure 7E). These results suggest that *yggS* contributes to the resistance of *A. citrulli* to BCDA.

### *yggS* Is Involved in HR Induction

**FIGURE 8:**
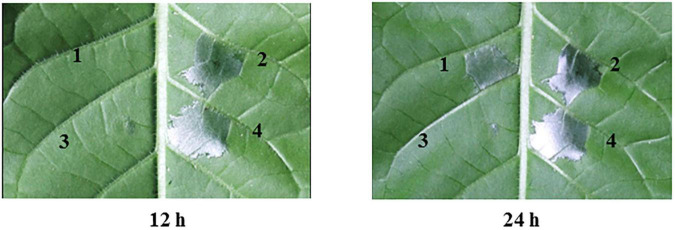
Effect of *yggS* on the ability of *Acidovorax citrulli* to induce a hypersensitive response (HR) on *Nicotiana tabacum*. All strains were cultured in LB overnight and infiltrated into the *N. tabacum* leaves at a concentration of OD_600_ = 0.3. *N. tabacum* was grown under greenhouse conditions at 24°C and observed for HR at 12 h and 24 h post infiltration. 1 = *yggS* deletion mutant Δ*yggS*, 2 = wild-type strain XjL12, 3 = negative control (NC) = double-distilled H_2_O (ddH_2_O), 4 = complemented strain C*yggS*.

To determine whether *yggS* contributes to the *A. citrulli* type III secretion system (T3SS), we examined HR induction by wild-type XjL12 and *yggS* mutant Δ*yggS* strains on *N. tabacum* (Figure 8). At 12 hpi, XjL12 and C*yggS* induced HR, while Δ*yggS* showed no cell death (similar to the negative control). However, HR induction was observed for Δ*yggS* at 24 hpi. These observations suggest that the deletion of *yggS* delays HR induction on *N. tabacum* and the deletion of *yggS* potentially impairs the T3SS function. In addition, the effect of reduced growth in Δ*yggS* on HR induction is inevitable.

### RNA-Seq Analysis Revealed That *yggS* Is Involved in the Regulation of T3SS *in vitro*

To investigate the cause of attenuated virulence observed for Δ*yggS*, the differences in the transcriptomes between Δ*yggS* and XjL12 were analyzed by RNA-Seq. A list containing the expression of all genes including the differentially expressed genes (DEGs) is provided in Supplementary File 2. The RNA-Seq results were validated by qPCR assay with ten chosen DEGs (Supplementary Figure 4). Compared with wild-type strain, there were 971 DEGs in the mutant strain: 506 genes were upregulated and 465 were downregulated (Supplementary Figure 5). In agreement with the previous abovementioned phenotypes, the DEGs were involved with *A. citrulli* motility, T3SS and anti-oxidative stress (Table 1).

**TABLE 1:** Differentially expressed genes related to *Acidovorax citrulli* pathogenicity.

Gene Id	log_2_FC	Description	*p* value
***T2SS***			
*Aave_2725*	−0.68704	type II secretion pathway component PulD-like protein	1.21E-07
*Aave_2721*	−0.55174	type II secretion system F family protein	0.000568
*Aave_4150*	0.27564	type II secretion system secretin GspD	0.007403
***T3SS***			
*Aave_0444*	−0.57401	AraC family transcriptional regulator HrpX (Zhang et al., 2018)	4.94E-05
*Aave_0468*	−2.1809	HrpB1 family type III secretion system apparatus protein	9.42E-20
*Aave_0479*	−1.2729	HrpB1 family type III secretion system apparatus protein	1.09E-05
*Aave_0464*	−1.4722	HrpE/YscL family type III secretion apparatus	6.38E-10
*Aave_0446*	−0.89909	type III secretion regulatory protein HpaB	0.000252
*Aave_0447*	−1.514	type III secretion regulatory protein HpaA	1.91E-21
*Aave_0463*	−1.0533	type III secretion system ATPase HrcN	7.86E-12
*Aave_3948*	−0.62563	type III secretion system chaperone	0.000666
*Aave_0450*	−2.6199	type III secretion system cytoplasmic ring protein HrcQ	5.92E-20
*Aave_0449*	−1.8577	type III secretion system export apparatus subunit HrcR	5.78E-08
*Aave_0452*	−2.1077	type III secretion system export apparatus subunit HrcV	8.67E-14
*Aave_0466*	−1.6779	type III secretion inner membrane ring lipoprotein HrcJ	3.32E-09
*Aave_0474*	−3.1228	type III secretion system outer membrane ring subunit HrcC	1.08E-21
***T6SS***			
*Aave_0497*	−0.75883	type VI secretion system tip protein VgrG	1.73E-14
*Aave_4009*	−0.4222	type VI secretion system tip protein VgrG	0.000394
***Oxidative stress***			
*Aave_0348*	0.30046	Peroxiredoxin	0.002919
*Aave_1172*	0.47563	peroxide stress protein YaaA	4.60E-05
*Aave_1235*	0.31254	Peroxiredoxin	0.002603
*Aave_1375*	0.33467	peroxiredoxin	0.000697
*Aave_1376*	0.40729	alkyl hydroperoxide reductase subunit F	0.000155
*Aave_2599*	−1.6937	superoxide dismutase family protein	2.11E-27
*Aave_3047*	0.56971	oxidative damage protection protein	3.57E-05
*Aave_3991*	−1.2794	Catalase	3.37E-16
*Aave_4272*	−1.2075	organic hydroperoxide resistance protein	2.35E-07
***Motility***			
*Aave_4418*	0.29599	flagellar biosynthesis anti-sigma factor FlgM	0.005056
*Aave_4679*	0.50058	pilin, PilA	1.85E-07
***QS***			
*Aave_3811*	−0.55673	acyl homoserine lactone synthase LuxI	0.003906

*Differentially expressed genes (DEGs) related to secretion system (T2SS, T3SS, and T6SS), oxidative stress, motility, quorum sensing (QS) in ΔyggS compared to wild-type strain XjL12 were listed in the table. Gene Id: the locus tags of DEGs that identified by hits in a Blastn search against the A. citrulli AAC00-1 genome (NC_008752). FC: fold change.*

Among these DEGs listed in Table 1, *in vitro* condition, a variety of T3SS genes including that encode indispensable apparatus subunit HrcC, HrcJ, et al., transcriptional regulator HrpX directly mediating the expression of most T3SS and T3Es genes significantly lowly-expressed. Correspondingly, the expressing level of many T3Es genes was decreased (Table 2). In addition to the T3Es revealed by annotation of genome, we also summarized the genes with high similarity to known T3Es from other plant pathogens based on the results reported by Jiménez-Guerrero et al. (2020). The homologues of *Aave_2177*, *Aave_1555*, *Aave_1244*, and *Aave_3960* have been validated as T3Es in *A. citrulli* strain M6 by translocation assay (Jiménez-Guerrero et al., 2020). Gene ontology (GO) analysis showed that down-regulated DEGs were enriched in terms related to secretion system (e.g., protein secretion, peptide secretion, secretion by cell) and regulations of these pathways were downregulated significantly in Δ*yggS* (Supplementary Figure 6A). Based on these factors, it is possible that YggS regulates a variety of pathogenic factors, and that regulation of secretion systems, especially T3SS, contributes to attenuated virulence on melon.

**TABLE 2:** Differentially expressed genes related to type III-secreted effectors.

Gene Id	log_2_FC	Description	*p* value
*Aave_0454* ^a^	−1.7491	type III secretion protein	0.009104
*Aave_0465* ^a^	−1.7458	type III secretion protein	5.26E-13
*Aave_0467* ^a^	−1.611	type III secretion protein	2.01E-07
*Aave_0473* ^a^	−1.8826	type III secretion protein	1.37E-28
*Aave_2166* ^a^	−0.52149	type III secretion system YopJ family effector	0.000437
*Aave_3621* ^a^	−0.29943	type III effector AopN (Zhang et al., 2020b)	0.006845
*Aave_3452* ^a^	−2.1122	hypothetical protein	2.25E-15
*Aave_4728* ^a^	−1.6464	hypothetical protein	1.94E-09
*Aave_3462* ^a^	−0.84509	peptidase C55	6.10E-06
*Aave_2177* ^b^	−1.6842	hypothetical protein	0.002479
*Aave_1555* ^b^	−1.1729	hypothetical protein	5.49E-30
*Aave_1244* ^b^	−0.97312	hypothetical protein	1.31E-12
*Aave_3960* ^b^	−0.92878	hypothetical protein	6.16E-07
*Aave_1090* ^c^	−1.7257	hypothetical protein	1.22E-27
*Aave_4254* ^c^	−1.6907	hypothetical protein	8.08E-13
*Aave_0458* ^c^	−1.6642	ribonuclease inhibitor	4.84E-09
*Aave_4612* ^c^	−1.554	hypothetical protein	6.88E-13
*Aave_4631* ^c^	−1.1544	hypothetical protein	2.41E-15
*Aave_4632* ^c^	−1.1128	leucine-rich repeat domain-containing protein	6.28E-21
*Aave_3847* ^c^	−0.63486	hypothetical protein	3.39E-07
*Aave_2802* ^c^	−0.43537	hypothetical protein	0.000197
*Aave_0310* ^c^	−0.35704	hypothetical protein	0.001025
*Aave_3621* ^c^	−0.29943	hypothetical protein	0.006845

*Differentially expressed genes (DEGs) related to putative type III-secreted effectors (T3Es) compared to wild-type strain XjL12 were listed in the table. Gene Id: the locus tags of DEGs that identified by hits in a Blastn search against the A. citrulli AAC00-1 genome (NC_008752). FC: fold change.*

*^a^The DEGs whose products are annotated as T3Es in A. citrulli strain AAC00-1 (Eckshtain-Levi et al., 2014).*

*^b^The homologues of these hypothetical proteins have been validted as T3Es in A. citrulli strain M6 by experimental assay (Jiménez-Guerrero et al., 2020).*

*^c^The gene product is similar to known T3Es by BlastP analyse (Jiménez-Guerrero et al., 2020).*

During infection, pathogens encounter the large generation of reactive oxygen species as part of the oxidative burst associated with plant defense response, making response to and protection against oxidative stress an important aspect for infection (Apel and Hirt, 2004; Burbank and Roper, 2014). The DEGs related to oxidative stress response partly listed in Table 1 indicated the perturbation in antioxidative response as a result of *yggS* mutation. FlgM and PilA have been conformed as a key factor responsible for flagellum and TFP assembly, respectively, in *A. citrulli* (Rosenberg et al., 2018; Yang et al., 2018), and both were upregulated in mutant (Table 1). Surprisingly, a gene *Aave_3811*, accounting for synthesizing acyl homoserine lactones-QS signal molecules (Fan et al., 2011), were negatively regulated. The expressing level of genes related to T2SS and T6SS was also changed in Δ*yggS*.

Ribosomes carrying out protein synthesis is required for cell growth (Lempiäinen and Shore, 2009). However, the biosynthesis of ribosomes is energy-consuming, thus the overproduction of ribosomal proteins is detrimental to cell proliferation (Jorgensen et al., 2004). GO analysis and KEGG pathway analysis revealed that the ribosome was most affected in *yggS* mutation (Supplementary Figures 6, 7). Supplementary Table 3 displayed the expression profile of the DEGs encoding 30S or 50S ribosomal proteins and all the genes were upregulated, indicating that inactive *yggS* caused increasing ribosomal biosynthesis, which may result in the growth defect. Previous reports have demonstrated the *yggS*-deficient *E. coil* impacts amino acids homeostasis, such as valine and isoleucine (Ito et al., 2013, 2019). According to KEGG pathway analysis, the pathway in valine, leucine and isoleucine degradation, β-Alanine metabolism was significantly regulated by *yggS*. In addition to both pathways, Supplementary Table 4 displayed the expression profile of the DEGs related to arginine and proline metabolism, alanine, aspartate and glutamate metabolism, glycine, serine and threonine metabolism, indicating that *yggS* also was involved in amino acids homeostasis in *A. citrulli*.

In addition, among the most GO enriched terms, only up-regulated DEGs were divided into terms belonging to cellular component domain, such as cellular anatomical entity, intracellular organelle, et al. (Supplementary Figure 6B). YggS appears to negatively affect pathway terms related to cellular component in *A. citrulli*.

## Discussion

YggS is a member of the highly conserved PLP-binding protein family classified as fold-type III family of PLP-dependent enzymes (Ito et al., 2019), which accounts for ∼4% of all activities classified by the Enzyme Commission (Percudani and Peracchi, 2003). PLP is involved in over 140 chemical reactions (Mooney et al., 2009) and is required for survival and virulence for some pathogens (Dick et al., 2010; Grubman et al., 2010; Xie et al., 2017). However, the role of YggS, which maintains PLP homeostasis, in pathogenicity remains poorly understood. To the best of our knowledge, this is first study of YggS in plant pathogenic bacteria and the first confirmation of its role in growth, secretion system, motility, oxidative stress response and virulence in *A. citrulli*.

A previous study indicated that high concentration of intracellular PNP is the root cause of partly pleiotropic phenotypes, e.g., toxicity of PN to *yggS* mutants and disordered amino acid metabolism (Ito et al., 2019). In the *yggS*-deficient strain, further accumulation of PNP induced by excess PN impacts the isoleucine/valine biosynthetic pathway, resulting in overproduction of valine, which is toxic to the cell. Because of a similar inhibitory effect of PN caused by *yggS*, it is possible that PNP was accumulated in the Δ*yggS*. Different from the reports in *E. coil* and *S. elongate* (Prunetti et al., 2016; Labella et al., 2017), in the case of *A. citrulli*, PL failed to suppress the toxicity of PN, but made up for the shortage in growth. The mechanism by which PL restore the Δ*yggS* growth needs to be studied in further detail.

The antibiotics DCS and BCDA are both peptidoglycan inhibitors that target alanine racemase, which is involved in the formation of D-alanine (Manning et al., 1974; David, 2001; Feng and Barletta, 2003). In contrast to the previous report on *S. elongate* (Labella et al., 2017), only sensitivity to BCDA was affected by deletion of *yggS* in *A. citrulli*. This difference may be because the targets of BCDA are not only alanine racemase, but also glutamate racemase (Prosser et al., 2016). In agreement with the observation described by Labella et al. (2017) in *S. elongate*, the sensitivity to BCDA caused by inactive *yggS* could be suppressed by L-alanine and D-alanine in *A. citrulli*, indicating that YggS may play a key role in preventing BCDA from destroying the activity of targets by altering the metabolism of alanine in the cell. It is worth mentioning that the overproduction of alanine racemase confers the resistance to DCS and BCDA (Cáceres et al., 1997; Feng and Barletta, 2003). However, we did not observe resistance when YggS was overexpressed in *A. citrulli* (Figure 7E and Supplementary Figure 3). This result indirectly indicates that YggS may have no racemase activity in *A. citrulli* despite showing structural similarity to alanine racemase, which is in consistent with the report in *E. coil* (Ito et al., 2013).

Based on a series of virulence assays, we conclude that *yggS* is indispensable for *A. citrulli* virulence including seed-to-seedling transmission and melon tissue colonization. To elucidate the role of YggS in pathogenicity, several factors associated with YggS were determined and these phenotypes were supported by genome-wide expression analysis. Additionally, according to RNA-Seq DEGs, YggS also regulates *A. citrulli* QS, however, this result needs to be validated in more detail in subsequent studies.

Previous studies reported a crucial role of vitamin B6 in protecting cells from oxidative stress (Mooney et al., 2009; Vanderschuren et al., 2013). For *Cercospora nicotianae* and *Rhizoctonia solani*, blocking vitamin B_6_ synthesis increased sensitivity to oxidative stress (Ehrenshaft et al., 1998; Samsatly et al., 2015). In addition, an *Actinobacillus pleuropneumoniae* mutant deficient in PLP synthase was sensitive to H_2_O_2_ and showed attenuated virulence (Xie et al., 2017). In the current study, increased sensitivity of Δ*yggS* to H_2_O_2_ demonstrated that YggS protects cell against oxidative stress. This can be explained by the result of the DEG analysis that showed genes related to oxidative stress response were differently expressed (Table 1). Based on the point that the level of PLP is controlled by YggS, it is possible that the normal oxidative stress response is impaired by disordered PLP or vitamin B_6_ levels in Δ*yggS*.

In swimming assay, loss or overexpression of *yggS* reduced swimming motility (Figure 6A). According to transcriptome analysis, the expression of anti-sigma factor FlgM, a negative factor for flagellar assembly (Frisk et al., 2002), was increased and the overexpression of FlgM may lead to reduced swimming. In addition, consistent with reduced swimming, flagella-related genes (*fliA*, *fliC*, *fliS*) in XjL12 (pYggS) were downregulated (Supplementary Figure 8). Nonetheless, the effect of molecular manipulation in overexpression strain could not been excluded. These findings suggest that the YggS level maybe critical to *A. citrulli* swimming motility. Based on the reports that the regulation to flagellar motility is PLP-dependent in pathogens *Helicobacter pylori* and *Campylobacter jejuni* (Dick et al., 2010; Asakura et al., 2013), we postulate that the weakened swimming may be due to unbalanced PLP level caused by defective YggS. However, we failed to recover the swimming ability by complementation vector pBBR-*yggS*. Perhaps the level of YggS in C*yggS* was less optimal than in the wild type strain. On the other hand, the increased expression of *pilA* may imply the enhanced twitching caused by inactive YggS. Moreover, *yggS* is not involved in *A. citrulli* biofilm formation (Supplementary Figure 9), which is closely related to pathogenicity for pathogenic bacteria.

It is widely accepted that T3SS is a pivotal mechanism for many gram-negative bacteria infecting host plants and eliciting HR on non-host plants (Mudgett, 2005). *A. citrulli*, possessing a *hrp* gene cluster, relies on a functional T3SS for pathogenicity (Johnson et al., 2011; Eckshtain-Levi et al., 2014). Therefore, in order to enhance screening efficiency, a Tn5 mutant library was screened by inoculating tobacco leaves. In this study, delayed HR phenotype induced by inactive *yggS* suggests that the function of T3SS was impaired. In agreement with delayed HR, T3SS genes related to core subunits of T3SS apparatus, T3SS regulators, T3SS chaperone and effectors showed reduced expression *in vitro*. Among these DEGs, genes for assembling T3SS in *A. citrulli* including *hrcC*, *hrcJ*, *hrcR* and *hrcV*, an AraC-type transcriptional regulator HrpX and effector AopN were confirmed to be necessary for *A. citrulli* to exert pathogenicity or elicit HR (Bahar and Burdman, 2010; Burdman and Walcott, 2012; Zhang et al., 2018, 2020b). Gene products that comprise T3SS can be grouped into four classes: apparatus proteins, translocon proteins, effectors and type III chaperones (Bronstein et al., 2000). According to the established genome annotations in *A. citrulli*, DEGs covered three classes except for translocon proteins. Therefore, we postulate that the compromised virulence is due to impaired function of T3SS. However, mechanism by which YggS regulates T3SS is unclear and to date, and no studies report the relationship of PLP and T3SS. Additionally, the further study on the association between *yggS* and T3SS-related genes or effectors *in planta* needs to be carried out.

In summary, we identified a novel pathogenicity-associated factor YggS that is required for *A. citrulli* virulence and involved in motility, secretion, antibiotic resistance, oxidative stress response and growth, especially in T3SS when cultured in LB medium. Based on the existing literature on YggS or its homolog along with our *in vitro* study results, we are unable to elucidate the precise mechanism by which YggS regulates virulence. Perhaps, the imbalance of PLP homeostasis or amino acid metabolism caused by the deficiency of YggS results in reduced virulence. Finally, our finding uncovers a broader function of YggS and provides new insights into the pathogenesis of *A. citrulli*.

## Conclusion

YggS, a pyridoxal 5′-phosphate binding protein that is conserved among the multiple organisms, is firstly reported as an indispensable factor for the virulence of *A. citrulli*, the causal agent of bacterial fruit blotch of cucurbits. The absence of YggS in *A. citrulli* reduces swimming motility, increases the sensitivity to H_2_O_2_, antibiotic BCDA and PN, and delays HR induction on *N. tabacum*. The RNA-Seq indicates that inactive YggS significantly impairs T3SS function and effectors translocation *in vitro*. The molecular details in the interaction between YggS and T3SS will be explored in the further study.

## Data Availability Statement

The datasets presented in this study can be found in online repositories. The names of the repository/repositories and accession number(s) can be found in the article/Supplementary Material.

## Author Contributions

YW, YT, and BH designed the experiments. YW, YZ, LC, BC, LX, WG, and YL performed the experiments and analyzed the data. YW wrote the manuscript. YZ, YT, and BH revised the manuscript. YT and BH provided guidance for the experiments. All authors contributed to the article and approved the submitted version.

## Conflict of Interest

The authors declare that the research was conducted in the absence of any commercial or financial relationships that could be construed as a potential conflict of interest.

## Publisher’s Note

All claims expressed in this article are solely those of the authors and do not necessarily represent those of their affiliated organizations, or those of the publisher, the editors and the reviewers. Any product that may be evaluated in this article, or claim that may be made by its manufacturer, is not guaranteed or endorsed by the publisher.

## Footnotes

1
https://bioinfo.ut.ee/primer3-0.4.0/

2
http://www.softberry.com/berry.phtml?topic=bprom&group=programs&subgroup=gfindb

## References

[B1] Apel K. Hirt H. (2004). Reactive oxygen species: metabolism, oxidative stress, and signal transduction. *Annu. Rev. Plant Biol.* 55 373–399. 10.1146/annurev.arplant.55.031903.141701 15377225

[B2] Araújo D. Mariano R. Michereff S. (2005). Inoculation methods of *Acidovorax avenae* subsp. *citrulli* in melon. *Summa Phytopathol*. 31 69–73.

[B3] Asakura H. Hashii N. Uema M. Kawasaki N. Sugita-Konishi Y. Igimi S. (2013). *Campylobacter jejuni pdxA* affects flagellum-mediated motility to alter host colonization. *PLoS one* 8:e70418. 10.1371/journal.pone.0070418 23936426 PMC3735588

[B4] Bahar O. Burdman S. (2010). Bacterial fruit blotch: a threat to the cucurbit industry. *Isr*. *J*. *Plant Sci*. 58 19–31. 10.1560/IJPS.58.1.19

[B5] Bahar O. Kritzman G. Burdman S. (2009a). Bacterial fruit blotch of melon: screens for disease tolerance and role of seed transmission in pathogenicity. *Eur*. *J*. *Plant Pathol*. 123 71–83. 10.1007/s10658-008-9345-7

[B6] Bahar O. Goffer T. Burdman S. (2009b). Type IV Pili are required for virulence, twitching motility, and biofilm formation of *Acidovorax avenae* subsp. *citrulli*. *Mol*. *Plant Microbe*. *Interact*. 22 909–920. 10.1094/MPMI-22-8-0909 19589067

[B7] Bahar O. Levi N. Burdman S. (2011). The cucurbit pathogenic bacterium *Acidovorax citrulli* requires a polar flagellum for full virulence before and after host-tissue penetration. *Mol*. *Plant Microbe*. *Interact*. 24 1040–1050. 10.1094/MPMI-02-11-0041 21554180

[B8] Bjarko M. E. Line R. F. (1988). Heritability and number of genes controlling leaf rust resistance in four cultivars of wheat. *Phytopathology* 78 457–461. 10.1094/Phyto-78-457

[B9] Bronstein P. A. Miao E. A. Miller S. I. (2000). InvB is a type III secretion chaperone specific for SspA. *J*. *Bacteriol*. 182 6638–6644. 10.1128/JB.182.23.6638-6644.2000 11073906 PMC111404

[B10] Burbank L. Roper M. C. (2014). OxyR and SoxR modulate the inducible oxidative stress response and are implicated during different stages of infection for the bacterial phytopathogen *Pantoea stewartii* subsp. *stewartii*. *Mol. Plant Microbe. Interact.* 27 479–490. 10.1094/MPMI-11-13-0348-R 24450773

[B11] Burdman S. Walcott R. (2012). *Acidovorax citrulli*: generating basic and applied knowledge to tackle a global threat to the cucurbit industry. *Mol*. *Plant Pathol*. 13 805–815. 10.1111/j.1364-3703.2012.00810.x 22738439 PMC6638624

[B12] Cáceres N. E. Harris N. B. Wellehan J. F. Feng Z. Kapur V. Barletta R. G. (1997). Overexpression of the D-alanine racemase gene confers resistance to D-cycloserine in *Mycobacterium smegmatis*. *J*. *Bacteriol*. 179 5046–5055. 10.1128/jb.179.16.5046-5055.1997 9260945 PMC179361

[B13] Chaban B. Hughes H. V. Beeby M. (2015). The flagellum in bacterial pathogens: For motility and a whole lot more. *Semin*. *Cell Dev*. *Biol*. 46 91–103. 10.1016/j.semcdb.2015.10.032 26541483

[B14] Darin N. Reid E. Prunetti L. Samuelsson L. Husain R. A. Wilson M. (2016). Mutations in PROSC disrupt cellular pyridoxal phosphate homeostasis and cause vitamin-B_6_-dependent epilepsy. *Am*. *J*. *Hum*. *Genet*. 99 1325–1337. 10.1016/j.ajhg.2016.10.011 27912044 PMC5142116

[B15] David S. (2001). Synergic activity of D-cycloserine and beta-chloro-D-alanine against *Mycobacterium tuberculosis*. *J*. *Antimicrob*. *Chemother*. 47 203–206. 10.1093/jac/47.2.203 11157908

[B16] Dick T. Manjunatha U. Kappes B. Gengenbacher M. (2010). Vitamin B6 biosynthesis is essential for survival and virulence of *Mycobacterium tuberculosis*. *Mol*. *Microbiol*. 78 980–988. 10.1111/j.1365-2958.2010.07381.x 20815826

[B17] Eckshtain-Levi N. Munitz T. Živanović M. Traore S. M. Spröer C. Zhao B. (2014). Comparative analysis of type III secreted effector genes reflects divergence of *Acidovorax citrulli* strains into three distinct lineages. *Phytopathology* 104 1152–1162. 10.1094/PHYTO-12-13-0350-R 24848275

[B18] Ehrenshaft M. Jenns A. E. Chung K. R. Daub M. E. (1998). *SOR1*, a gene required for photosensitizer and singlet oxygen resistance in *Cercospora* fungi, is highly conserved in divergent organisms. *Mol*. *Cell* 1 603–609. 10.1016/S1097-2765(00)80060-X 9660944

[B19] Eswaramoorthy S. Gerchman S. Graziano V. Kycia H. Studier F. W. Swaminathan S. (2003). Structure of a yeast hypothetical protein selected by a structural genomics approach. *Acta Crystallogr*. *D*. *Biol*. *Crystallogar*. 59 127–135. 10.1107/S0907444902018012 12499548

[B20] Fan J. Qian G. Chen T. Zhao Y. Liu F. Walcott R. R. (2011). The acyl-homoserine lactone (AHL)-type quorum sensing system affects growth rate, swimming motility and virulence in *Acidovorax avenae* subsp *citrulli*. *World J*. *Microbiol*. *Biotechnol*. 27 1155–1166. 10.1007/s11274-010-0562-9

[B21] Feng F. Zhou J. M. (2012). Plant-bacterial pathogen interactions mediated by type III effectors. *Curr*. *Opin*. *Plant Biol*. 15 469–476. 10.1016/j.pbi.2012.03.004 22465133

[B22] Feng Z. Barletta R. G. (2003). Roles of *Mycobacterium smegmatis* D-alanine:D-alanine ligase and D-alanine racemase in the mechanisms of action of and resistance to the peptidoglycan inhibitor D-cycloserine. *Antimicrob*. *Agents Chemother*. 47 283–291. 10.1128/AAC.47.1.283-291.2003 12499203 PMC149019

[B23] Frisk A. Jyot J. Arora S. K. Ramphal R. (2002). Identification and functional characterization of *flgM*, a gene encoding the anti-sigma 28 factor in *Pseudomonas aeruginosa*. *J*. *Bacteriol*. 184 1514–1521. 10.1128/JB.184.6.1514-1521.2002 11872701 PMC134903

[B24] Ge Y. Luo L. Xia L. Luo X. Bi H. Gong H. (2021). Fermentation: an unreliable seed treatment for bacterial fruit blotch of watermelon. *Plant Dis*. 105 1026–1033. 10.1094/PDIS-05-20-1056-RE 33507094

[B25] Grubman A. Phillips A. Thibonnier M. Kaparakis-Liaskos M. Johnson C. Thiberge J. M. (2010). Vitamin B_6_ is required for full motility and virulence in *Helicobacter pylori*. *mBio* 1:e00112-10. 10.1128/mBio.00112-10 21151756 PMC3000542

[B26] Hopkins D. L. Thompson C. M. (2002). Seed transmission of *Acidovorax avenae* subsp. *citrulli* in cucurbits. *HortScience* 37:924. 10.1023/A:1020855405948

[B27] Ito T. Iimori J. Takayama S. Moriyama A. Yamauchi A. Hemmi H. (2013). Conserved pyridoxal protein that regulates Ile and Val metabolism. *J*. *Bacteriol*. 195 5439–5449. 10.1128/JB.00593-13 24097949 PMC3889608

[B28] Ito T. Yamamoto K. Hori R. Yamauchi A. Downs D. M. Hemmi H. (2019). Conserved pyridoxal 5′-phosphate-binding protein YggS impacts amino acid metabolism through pyridoxine 5′-phosphate in *Escherichia coli*. *Appl*. *Environ*. *Microbiol*. 85:e00430-19. 10.1128/AEM.00430-19 30902856 PMC6532037

[B29] Ito T. Yamauchi A. Hemmi H. Yoshimura T. (2016). Ophthalmic acid accumulation in an *Escherichia coli* mutant lacking the conserved pyridoxal 5′-phosphate-binding protein YggS. *J*. *Biosci*. *Bioeng*. 122 689–693. 10.1016/j.jbiosc.2016.06.010 27426274

[B30] Jiménez-Guerrero I. Perez-Montano F. Da Silva G. M. Wagner N. Shkedy D. Zhao M. (2020). Show me your secret(ed) weapons: a multifaceted approach reveals a wide arsenal of type III-secreted effectors in the cucurbit pathogenic bacterium *Acidovorax citrulli* and novel effectors in the *Acidovorax* genus. *Mol*. *Plant Pathol*. 21 17–37. 10.1111/mpp.12877 31643123 PMC6913199

[B31] Johnson K. L. Minsavage G. V. Walcott R. R. (2009). Effect of type III and type II secretion on *Acidovorax avenae* subsp *citrulli* colonization of watermelon seed and seedling tissue. *Phytopathology* 99 S59–S59.

[B32] Johnson K. L. Minsavage G. V. Le T. Jones J. B. Walcott R. R. (2011). Efficacy of a nonpathogenic *Acidovorax citrulli* strain as a biocontrol seed treatment for bacterial fruit blotch of cucurbits. *Plant Dis*. 95 697–704. 10.1094/PDIS-09-10-0660 30731899

[B33] Johnstone D. L. Al-Shekaili H. H. Tarailo-Graovac M. Wolf N. I. Ivy A. S. Demarest S. (2019). PLPHP deficiency: clinical, genetic, biochemical, and mechanistic insights. *Brain* 142 542–559. 10.1093/brain/awy346 30668673 PMC6391652

[B34] Jorgensen P. Tyers M. Warner J. R. (2004). “Forging the factory: ribosome synthesis and growth control in budding yeast,” in *Cell Growth: Control of Cell Size*, eds Hall M. N. Raff M. Thomas G. (Cold Spring Harbor, NY: Laboratory Press).

[B35] Kim M. Lee J. Heo L. Han S. W. (2020). Putative bifunctional chorismate mutase/prephenate dehydratase contributes to the virulence of *Acidovorax citrulli*. *Front*. *Plant Sci*. 11:569552. 10.3389/fpls.2020.569552 33101336 PMC7546022

[B36] Labella J. I. Cantos R. Espinosa J. Forcada-Nadal A. Rubio V. Contreras A. (2017). PipY, a member of the conserved COG0325 family of PLP-binding proteins, expands the cyanobacterial nitrogen regulatory network. *Front*. *Microbiol*. 8:1244. 10.3389/fmicb.2017.01244 28744260 PMC5504682

[B37] Lempiäinen H. Shore D. (2009). Growth control and ribosome biogenesis. *Curr. Opin. Cell Biol.* 6 855–863. 10.1016/j.ceb.2009.09.002 19796927

[B38] Liu J. Luo S. Z. Zhang Q. Wang Q. H. Chen J. F. Guo A. G. (2012). Tn5 transposon mutagenesis in *Acidovorax citrulli* for identification of genes required for pathogenicity on cucumber. *Plant Pathol*. 61 364–374. 10.1111/j.1365-3059.2011.02519.x

[B39] Liu J. Tian Y. Zhao Y. Zeng R. Chen B. Hu B. (2019). Ferric uptake regulator (FurA) is required for *Acidovorax citrulli* virulence on watermelon. *Phytopathology* 109 1997–2008. 10.1094/PHYTO-05-19-0172-R 31454303

[B40] Livak K. J. Schmittgen T. D. (2001). Analysis of relative gene expression data using real-time quantitative PCR and the 2(-Delta Delta C(T)) method. *Methods* 25 402–408. 10.1006/meth.2001 11846609

[B41] Macho A. P. Zipfel C. (2015). Targeting of plant pattern recognition receptor-triggered immunity by bacterial type-III secretion system effectors. *Curr*. *Opin*. *Microbiol*. 23 14–22. 10.1016/j.mib.2014.10.009 25461568

[B42] Manning J. M. Merrifield N. E. Jones W. M. Gotschlich E. C. (1974). Inhibition of bacterial growth by beta-chloro-D-alanine. *Proc*. *Natl*. *Acad*. *Sci*. *U.S.A*. 71 417–421. 10.1073/pnas.71.2.417 4150023 PMC388017

[B43] Mattick J. S. (2002). Type IV pili and twitching motility. *Annu*. *Rev*. *Microbiol*. 56 289–314. 10.1146/annurev.micro.56.012302.160938 12142488

[B44] Mooney S. Leuendorf J. E. Hendrickson C. Hellmann H. (2009). Vitamin B6: A long known compound of surprising complexity. *Molecules* 14 329–351. 10.3390/molecules14010329 19145213 PMC6253932

[B45] Mudgett M. B. (2005). New insights to the function of phytopathogenic bacterial type III effectors in plants. *Annu*. *Rev*. *Plant Biol*. 56 509–531. 10.1146/annurev.arplant.56.032604.144218 15862106

[B46] Percudani R. Peracchi A. (2003). A genomic overview of pyridoxal-phosphate-dependent enzymes. *EMBO Rep*. 4 850–854. 10.1038/sj.embor.embor914 12949584 PMC1326353

[B47] Pfeilmeier S. Caly D. L. Malone J. G. (2016). Bacterial pathogenesis of plants: future challenges from a microbial perspective: Challenges in bacterial molecular plant pathology. *Mol*. *Plant Pathol*. 17 1298–1313. 10.1111/mpp.12427 27170435 PMC6638335

[B48] Prosser G. A. Rodenburg A. Khoury H. de Chiara C. Howell S. Snijders A. P. (2016). Glutamate racemase is the primary target of β-chloro-d-alanine in Mycobacterium *tuberculosis*. *Antimicrob*. *Agents Chemother*. 60 6091–6099. 10.1128/AAC.01249-16 27480853 PMC5038272

[B49] Prunetti L. El Yacoubi B. Schiavon C. R. Kirkpatrick E. Huang L. Bailly M. (2016). Evidence that COG0325 proteins are involved in PLP homeostasis. *Microbiology* 162 694–706. 10.1099/mic.0.000255 26872910

[B50] Ren Z. G. Hou L. Song Z. G. Zhang L. Q. (2009). Screening of the pathogenicity mutants of *Acidovorax avenae* subsp. *citrulli* and cloning of *hrcR* gene. *Acta Phytopathol. Sin.* 39 501–506.

[B51] Rosenberg T. Salam B. B. Burdman S. (2018). Association between loss of type IV pilus synthesis ability and phenotypic variation in the cucurbit pathogenic bacterium *Acidovorax citrulli*. *Mol*. *Plant Microbe*. *Interact*. 31 548–559. 10.1094/MPMI-12-17-0324-R 29298127

[B52] Rusmini R. Vecchietti D. Macchi R. Vidal-Aroca F. Bertoni G. (2014). A shotgun antisense approach to the identification of novel essential genes in *Pseudomonas aeruginosa*. *BMC Microbiol*. 14:24. 10.1186/1471-2180-14-24 24499134 PMC3922391

[B53] Sambrook J. Fritsch E. F. Maniatis T. A. (1989). *Molecular Cloning: A Laboratory Manual*, 2nd Edn. Cold Spring Harbor, NY: Cold Spring Harbor Laboratory Press.

[B54] Samsatly J. Chamoun R. Gluck-Thaler E. Jabaji S. (2015). Genes of the de novo and salvage biosynthesis pathways of vitamin B_6_ are regulated under oxidative stress in the plant pathogen *Rhizoctonia solani*. *Front*. *Microbiol*. 6:1429. 10.3389/fmicb.2015.01429 26779127 PMC4700284

[B55] Schaad N. W. Postnikova E. Sechler A. Claflin L. E. Vidaver A. K. Jones J. B. (2008). Reclassification of subspecies of *Acidovorax avenae* as *A. Avenae* (Manns 1905) emend., *A. cattleyae* (Pavarino, 1911) comb. nov., *A. citrulli* Schaad et al., 1978) comb. nov., and proposal of *A. oryzae* sp. nov. *Syst*. *Appl*. *Microbiol*. 31 434–446. 10.1016/j.syapm.2008.09.003 18993005

[B56] Schaad N. W. Sowell G. Goth R. W. Colwell R. R. Webb R. E. (1978). *Pseudomonas pseudoalcaligenes* subsp. *citrulli* subsp. nov. *Int*. *J*. *Syst*. *Bacteriol*. 28 117–125. 10.1099/00207713-28-1-117

[B57] Tian Y. Zhao Y. Wu X. Liu F. Hu B. Walcott R. R. (2015). The type VI protein secretion system contributes to biofilm formation and seed-to-seedling transmission of *Acidovorax citrulli* on melon. *Mol*. *Plant Pathol*. 16 38–47. 10.1111/mpp.12159 24863458 PMC6638315

[B58] Tremino L. Forcada-Nadal A. Contreras A. Rubio V. (2017). Studies on cyanobacterial protein PipY shed light on structure, potential functions, and vitamin B_6_-dependent epilepsy. *FEBS Lett*. 591 3431–3442. 10.1002/1873-3468.12841 28914444

[B59] Vanderschuren H. Boycheva S. Li K. T. Szydlowski N. Gruissem W. Fitzpatrick T. B. (2013). Strategies for vitamin B6 biofortification of plants: a dual role as a micronutrient and a stress protectant. *Front*. *Plant Sci*. 4:143. 10.3389/fpls.2013.00143 23734155 PMC3659326

[B60] Vu H. N. Ito T. Downs D. M. (2020). The role of YggS in vitamin B_6_ homeostasis in *Salmonella enterica* is informed by heterologous expression of yeast SNZ3. *J*. *Bacteriol*. 202:e00383-20. 10.1128/JB.00383-20 32900833 PMC7585061

[B61] Walcott R. R. Gitaitis R. D. (2000). Detection of *Acidovorax avenae* subsp. *citrulli* in watermelon seed using immunomagnetic separation and the polymerase chain reaction. *Plant Dis*. 84 470–474. 10.1094/PDIS.2000.84.4.470 30841172

[B62] Wang T. Guan W. Huang Q. Yang Y. Yan W. Sun B. (2016). Quorum-sensing contributes to virulence, twitching motility, seed attachment and biofilm formation in the wild type strain Aac-5 of *Acidovorax citrulli*. *Microb*. *Pathog*. 100 133–140. 10.1016/j.micpath.2016.08.039 27594669

[B63] Webb R. E. Goth R. W. (1965). A seedborne bacterium isolated from watermelon. *Plant Dis*. *Rep*. 49 818–821.

[B64] Willems A. Goor M. Thielemans S. Gillis M. Kersters K. De Ley J. (1992). Transfer of several phytopathogenic *Pseudomonas* species to *Acidovorax* as *Acidovorax avenae* subsp. *avenae* subsp. nov., comb. nov., *Acidovorax avenae* subsp. *citrulli*, *Acidovorax avenae* subsp. *cattleyae*, and *Acidovorax konjaci*. *Int*. *J*. *Syst*. *Bacteriol*. 42 107–119. 10.1099/00207713-42-1-107 1371056

[B65] Xie F. Li G. Wang Y. Zhang Y. Zhou L. Wang C. (2017). Pyridoxal phosphate synthases PdxS/PdxT are required for *Actinobacillus pleuropneumoniae* viability, stress tolerance and virulence. *PLoS One* 12:e0176374. 10.1371/journal.pone.0176374 28448619 PMC5407770

[B66] Yang B. Y. Hu F. P. Cai X. Q. (2018). Function analysis of flagellin gene *flgM* in *Acidovorax citrulli*. *J. Agric. Biotechnol. (China)* 27 504–515. 10.3969/j.issn.1674-7968.2019.03.015

[B67] Zhang X. Yang Y. Zhao M. Yang L. Jiang J. Walcott R. (2020a). *Acidovorax citrulli* type III effector Aopp suppresses plant immunity by targeting the watermelon transcription factor WRKY6. *Front*. *Plant Sci*. 11:579218. 10.3389/fpls.2020.579218 33329640 PMC7718035

[B68] Zhang X. Zhao M. Jiang J. Yang L. Yang Y. Yang S. (2020b). Identification and functional analysis of AopN, an *Acidovorax citrulli* effector that induces programmed cell death in plants. *Int*. *J*. *Mol*. *Sci*. 21:6050. 10.3390/ijms21176050 32842656 PMC7504669

[B69] Zhang X. Zhao M. Yan J. Yang L. Yang Y. Guan W. (2018). Involvement of *hrpX* and *hrpG* in the virulence of *Acidovorax citrulli* strain Aac5, causal agent of bacterial fruit blotch in cucurbits. *Front*. *Microbiol*. 9:507. 10.3389/fmicb.2018.00507 29636729 PMC5880930

