# Supplementary material for: yggS Encoding Pyridoxal 5′-Phosphate Binding Protein Is Required for Acidovorax citrulli Virulence
Source: Front Microbiol. 2022 Jan 11;12:783862. doi: 10.3389/fmicb.2021.783862 (PMC8787154; doi:10.3389/fmicb.2021.783862)
Supplement: Supplementary file 1 [file Data_Sheet_1.DOCX]

***Supplementary Material***

**Table S1**. Strains and plasmids used in the study

| Strains or plasmids | Characteristics^a^ | Source or reference |
| --- | --- | --- |
| *Acidovorax citrulli* |  |  |
| XjL12 | Wild-type strain, Rif^R^ | This lab |
| sk24 | Tn5 mutant defective in *yggS* | This study |
| Δ*yggS* | *yggS* mutant strain, containing truncated *yggS* gene and Km cassette, Rif^R^, Km^R^ | This study |
| C*yggS* | complementation strain of Δ*yggS*, containing pBBR-*yggS*, Rif^R^, Km^R^, Gm^R^ | This study |
| XjL12(pYggS) | *yggS* overexpression in XjL12, containing pBBR-yggS, Rif^R^, Gm^R^ | This study |
| *Escherichia coli* |  |  |
| DH5α | *Φ80 lacZDM15*, *D(lacZYA-argF)U169*. *recA1*, *endA1*.*thi-1* | TaKaRa, Dalian, China |
| BW20676 | *Δpir pro hsdR*, *recA* | This lab |
| Plasmids |  |  |
| pEX18GM | Suicide vector with *sacB* gene, Gm^R^ | (Hoang et al. 1998) |
| pEX18*yggS*Km | Containing wild-type yggs gene replaced by Km cassette, Gm^R^, Km^R^ | This study |
| pBBR1-MCS-5 | Broad-host-range vector with a P*_lac_* Promoter, Gm^R^ | (Kovach et al. 1995) |
| pBBR-*yggS* | Open reading frame of yggS and its promoter region cloned into pBBR1-MCS-5, Gm^R^ | This study |
| pET30 | Containing kanamycin cassette, Km^R^ | This lab |
| pUT-Km | Delivery plasmid for Tn5; R6K rep licon; Amp^R^, Km^R^ | This lab |
| pRK600 | ColE1 ori V; RP4; tra^+^; RP4 oriT; helper plasmid in Triparental mating, Cm^R^ | This lab |
| pUC19 | ColE1 origin, Amp^R^ | This lab |

^a^ Rif^R^, Km^R^, Gm^R^, Amp^R^, Cm^R^ represent the resistance to rifamycin, kanamycin, gentamicin, ampicillin and chloramphenicol, respectively.

**References:**

Hoang, T. T., Karkhoff-Schweizer, R. R., Kutchma, A. J. and Schweizer, H. P. (1998). A broad-host-range Flp-FRT recombination system for site-specific excision of chromosomally-located DNA sequences: application for isolation of unmarked *Pseudomonas aeruginosa* mutants. *Gene* 212, 77-86. doi: 10.1016/s0378-1119(98)00130-9

Kovach, M. E., Elzer, P. H., Hill, D. S., Robertson, G. T., Farris, M. A., Roop, R. M., et al. (1995). Four new derivatives of the broad-host-range cloning vector pBBR1MCS, carrying different antibiotic-resistance cassettes. *Gene* 166, 175-176. doi: 10.1016/0378-1119(95)00584-1

**Table S2**. PCR and qPCR primers used in this study

| Primers | Sequence (5′ to 3′)^a^ | Length of production |
| --- | --- | --- |
| PCR primers |  |  |
| WFB1 | GACCAGCCACACACTGGGAC | 360 |
| WFB2 | CTGCCGTACTCCAGCGAT |  |
| Tn5-F | CTATCGATTGTATGGGAAGCC | 586 |
| Tn5-R | CGAGGCAGTTCCATAGGATGG |  |
| *yggS*-upF | CCAAGCTTGAGGATCTTGCTCGGGATGG | 467 |
| *yggS*-upR | GCTCTAGAGAAGGTCTTGGAAACGGCGA |  |
| *yggS*-downF | CGGGATCCGAGGACTTCGACACGCTCTC | 480 |
| *yggS*-downR | GGGGTACCCTATGCCCTCGACCACCAC |  |
| *yggS*-F | GGGGTACCATGTCCACGAAAAACCCTTG | 837 |
| *yggS*-R | CGGGATCCTCAGCGCGCCGGATAGCTGC |  |
| Km-F | GCTCTAGAGAAGCTCCCTCGTGC | 1514 |
| Km-R | CGGGATCCCAGGTGGCACTTTTCG |  |
| qPCR primers |  |  |
| *16s*-F | CCTACGGGAGGCAGCAG | 177 |
| *16s*-R | ATTACCGCGGCTGCTGG |  |
| *pilA*-F | GAACTGATGATCGTGGTGGC | 168 |
| *pilA*-R | CGGAGGACTCGAAGCAGTAG |  |
| *flgM*-F | CCGAAGAGGCCACCAAGACC | 117 |
| *flgM*-R | CATCGAAATCCGCGCTGCTG |  |
| *hrcC*-F | CCCGGTGGATGGCAAGAACA | 171 |
| *hrcC*-R | TCCGATTCGACGGTGATGCC |  |
| *hrcJ*-F | CCTGGAACGTGCAGGTGGAA | 170 |
| *hrcJ*-R | CTGGGCGACGCCATAGATGA |  |
| *hrcN*-F | CGTGGGCAAGAGTACGCTGA | 162 |
| *hrcN*-R | GAGGTGGCGCAGACCACTAC |  |
| *hrcQ*-F | CGAAGTGGCGGAAACCCTGA | 125 |
| *hrcQ*-R | CGCCCTGGGTCCATTCCAG |  |
| *hrcV*-F | TCCGGCTGTCGCTGAACATC | 167 |
| *hrcV*-R | GCCCTTGGCGATGACGATGA |  |
| *hrpX*-F | GAGTCGCTGCGGCAGTACA | 176 |
| *hrpX*-R | GGGTCGTGCAGGTTCTCGAT |  |
| *hrcR*-F | GCCCAACATGGTGCTCAACG | 177 |
| *hrcR*-R | TCCAGGAACTTGCGGAACGG |  |
| *hrpB1*-F | AGGCGCTGCAGTACCTGAAC | 177 |
| *hrpB1*-R | CGTCCTCGGGTTCCATCTGC |  |

^a^ Base sequences underlined indicated the restriction enzyme sites.

**Table S3.** List of the differentially expressed genes related to ribosome synthesis

| Gene Id | log_2_FC | Description | *p* value |
| --- | --- | --- | --- |
| *Aave_0333* | 0.64046 | 30S ribosomal protein S12 | 1.05E-08 |
| *Aave_0334* | 0.65778 | 30S ribosomal protein S7 | 6.16E-11 |
| *Aave_0337* | 1.3714 | 30S ribosomal protein S10 | 3.11E-38 |
| *Aave_0342* | 1.6728 | 30S ribosomal protein S19 | 7.40E-29 |
| *Aave_0344* | 1.4432 | 30S ribosomal protein S3 | 3.29E-22 |
| *Aave_0347* | 1.7244 | 30S ribosomal protein S17 | 2.16E-21 |
| *Aave_0614* | 1.2929 | 30S ribosomal protein S14 | 1.98E-14 |
| *Aave_0615* | 1.4385 | 30S ribosomal protein S8 | 1.47E-25 |
| *Aave_0618* | 1.5214 | 30S ribosomal protein S5 | 4.07E-32 |
| *Aave_0623* | 1.2812 | 30S ribosomal protein S13 | 9.55E-27 |
| *Aave_0624* | 1.1513 | 30S ribosomal protein S11 | 6.76E-19 |
| *Aave_0625* | 1.2023 | 30S ribosomal protein S4 | 6.42E-26 |
| *Aave_1167* | 1.1843 | 30S ribosomal protein S20 | 2.31E-05 |
| *Aave_1228* | 1.3211 | 30S ribosomal protein S6 | 1.75E-18 |
| *Aave_1230* | 1.7467 | 30S ribosomal protein S18 | 6.51E-21 |
| *Aave_1258* | 0.71075 | 30S ribosomal protein S15 | 2.19E-09 |
| *Aave_1645* | 0.35971 | 30S ribosomal protein S12 methylthiotransferase RimO | 0.000657 |
| *Aave_1823* | 0.60606 | 30S ribosomal protein S2 | 2.87E-09 |
| *Aave_1884* | 1.5672 | 30S ribosomal protein S16 | 2.76E-18 |
| *Aave_2092* | 1.402 | 30S ribosomal protein S21 | 5.62E-09 |
| *Aave_3285* | 0.92227 | 30S ribosomal protein S1 | 4.14E-18 |
| *Aave_3382* | 0.64445 | 30S ribosome-binding factor RbfA | 2.39E-09 |
| *Aave_4052* | 1.4044 | 30S ribosomal protein S9 | 1.31E-13 |
| *Aave_0338* | 0.69024 | 50S ribosomal protein L3 | 4.20E-08 |
| *Aave_0339* | 1.1185 | 50S ribosomal protein L4 | 8.54E-10 |
| *Aave_0341* | 1.3947 | 50S ribosomal protein L2 | 5.28E-24 |
| *Aave_0343* | 1.384 | 50S ribosomal protein L22 | 1.35E-24 |
| *Aave_0345* | 1.588 | 50S ribosomal protein L16 | 4.16E-21 |
| *Aave_0346* | 1.7276 | 50S ribosomal protein L29 | 6.29E-06 |
| *Aave_0611* | 0.95188 | 50S ribosomal protein L14 | 2.29E-18 |
| *Aave_0612* | 1.2119 | 50S ribosomal protein L24 | 7.46E-10 |
| *Aave_0613* | 1.2544 | 50S ribosomal protein L5 | 6.11E-15 |
| *Aave_0616* | 1.4804 | 50S ribosomal protein L6 | 1.73E-41 |
| *Aave_0617* | 1.5706 | 50S ribosomal protein L18 | 2.63E-28 |
| *Aave_0619* | 1.9296 | 50S ribosomal protein L30 | 8.63E-08 |
| *Aave_0620* | 1.4736 | 50S ribosomal protein L15 | 1.76E-39 |
| *Aave_0627* | 1.1112 | 50S ribosomal protein L17 | 1.86E-25 |
| *Aave_1231* | 1.6382 | 50S ribosomal protein L9 | 2.93E-23 |
| *Aave_1887* | 1.1276 | 50S ribosomal protein L19 | 1.31E-25 |
| *Aave_2856* | 1.0354 | 50S ribosomal protein L20 | 1.33E-25 |
| *Aave_2857* | 0.75959 | 50S ribosomal protein L35 | 2.68E-05 |
| *Aave_3428* | 1.3386 | 50S ribosomal protein L28 | 2.23E-08 |
| *Aave_3611* | 1.073 | 50S ribosomal protein L25/general stress protein Ctc | 6.99E-19 |
| *Aave_3674* | 1.3147 | 50S ribosomal protein L27 | 1.35E-13 |
| *Aave_3675* | 1.0484 | 50S ribosomal protein L21 | 2.76E-18 |
| *Aave_4053* | 1.3135 | 50S ribosomal protein L13 | 6.94E-20 |
| *Aave_4532* | 1.5657 | 50S ribosomal protein L7/L12 | 3.13E-23 |
| *Aave_4533* | 1.4081 | 50S ribosomal protein L10 | 5.53E-41 |
| *Aave_4534* | 0.94589 | 50S ribosomal protein L1 | 9.68E-19 |
| *Aave_4535* | 1.0287 | 50S ribosomal protein L11 | 2.51E-25 |

Note: Differentially expressed genes (DEGs) related to ribosomal protein biosynthesis in Δ*yggS* compared to wild-type strain XjL12 were listed in the table. Gene Id: the locus tags of DEGs that identified by hits in a Blastn search against the *A. citrulli* AAC00-1 genome (NC_008752). FC: fold change

**Table S4.** List of the differentially expressed genes related to amino acids metabolism

| Gene Id | log_2_FC | Description | *p* value |
| --- | --- | --- | --- |
| ***Valine, leucine and isoleucine degradation*** | | | |
| *Aave_0633* | -0.2614 | NAD(P)-dependent oxidoreductase | 0.008562 |
| *Aave_1515* | 0.369 | malonyl-CoA synthase | 0.002297 |
| *Aave_1652* | 1.8362 | hydroxymethylglutaryl-CoA lyase | 0.001455 |
| *Aave_2433* | -0.71851 | dihydrolipoyl dehydrogenase | 2.95E-09 |
| *Aave_2464* | -0.38819 | dihydrolipoyl dehydrogenase | 7.00E-05 |
| *Aave_2478* | -0.32694 | acetyl-CoA C-acetyltransferase | 0.000774 |
| *Aave_2510* | -0.40715 | methylmalonyl-CoA mutase | 4.14E-05 |
| *Aave_2513* | -0.43344 | acyl-CoA carboxylase subunit beta | 5.64E-05 |
| *Aave_2515* | -0.56181 | acetyl/propionyl/methylcrotonyl-CoA carboxylase subunit alpha | 2.21E-07 |
| *Aave_2600* | -1.9873 | 3-hydroxyisobutyrate dehydrogenase | 1.77E-71 |
| *Aave_2604* | -1.9741 | CoA-acylating methylmalonate-semialdehyde dehydrogenase | 4.45E-47 |
| *Aave_3126* | 0.66384 | CoA transferase subunit B | 0.006110 |
| *Aave_3127* | 0.53066 | CoA transferase subunit A | 0.005801 |
| *Aave_3510* | 0.53891 | ATP-grasp domain-containing protein | 0.002052 |
| *Aave_4744* | 1.3457 | aspartate aminotransferase family protein | 8.06E-07 |
| *Aave_4746* | 1.4847 | CoA-acylating methylmalonate-semialdehyde dehydrogenase | 0.000223 |
| ***Valine, leucine and isoleucine biosynthesis*** | | | |
| *Aave_0019* | -0.42705 | 2-isopropylmalate synthase | 9.88E-05 |
| *Aave_2263* | -0.27725 | thiamine pyrophosphate-binding protein | 4.20E-16 |
| *Aave_3108* | 0.25458 | 2-isopropylmalate synthase | 0.005163 |
| *Aave_3110* | 0.56479 | ketol-acid reductoisomerase | 0.008596 |
| *Aave_4441* | 1.2117 | threonine dehydratase | 5.58E-05 |
| ***beta-Alanine metabolism*** | | | |
| *Aave_0966* | 2.0412 | NAD(P)-dependent oxidoreductase | 5.85E-28 |
| *Aave_0967* | 2.2013 | NAD-dependent dihydropyrimidine dehydrogenase subunit PreA | 1.24E-17 |
| *Aave_0969* | 2.0365 | dihydropyrimidinase | 9.78E-21 |
| *Aave_1511* | -0.37525 | malonyl-CoA decarboxylase | 0.000165 |
| *Aave_2604* | -1.9741 | CoA-acylating methylmalonate-semialdehyde dehydrogenase | 4.45E-47 |
| *Aave_4744* | 1.3457 | aspartate aminotransferase family protein | 8.06E-07 |
| *Aave_4746* | 1.4847 | CoA-acylating methylmalonate-semialdehyde dehydrogenase | 0.000223 |
| ***Arginine and proline metabolism*** | | | |
| *Aave_0121* | -1.1439 | ornithine cyclodeaminase | 1.87E-06 |
| *Aave_0120* | -0.94668 | arginase | 0.001211 |
| *Aave_0191* | 0.39279 | amidase | 0.003579 |
| *Aave_0601* | 0.88316 | pyrroline-5-carboxylate reductase | 2.41E-11 |
| *Aave_3321* | 1.1857 | trifunctional transcriptional regulator/proline dehydrogenase/L-glutamate gamma-semialdehyde dehydrogenase | 0.000239 |
| ***Alanine, aspartate and glutamate metabolism*** | | | |
| *Aave_0188* | -1.4839 | Glu/Leu/Phe/Val dehydrogenase | 2.23E-18 |
| *Aave_1442* | 0.3759 | type I glutamate--ammonia ligase | 8.42E-05 |
| *Aave_1856* | 0.37706 | argininosuccinate synthase | 0.003173 |
| *Aave_1863* | 0.27551 | amidophosphoribosyltransferase | 0.009259 |
| *Aave_2610* | 0.31638 | glutamine-hydrolyzing carbamoyl-phosphate synthase small subunit | 0.001644 |
| *Aave_2611* | 0.94521 | carbamoyl-phosphate synthase large subunit | 2.67E-17 |
| *Aave_3321* | 1.1857 | trifunctional transcriptional regulator/proline dehydrogenase/L-glutamate gamma-semialdehyde dehydrogenase | 0.000239 |
| *Aave_4182* | 1.1167 | asparaginase | 4.34E-07 |
| ***Glycine, serine and threonine metabolism*** | | | |
| *Aave_1219* | 0.2904 | aspartate-semialdehyde dehydrogenase | 0.005262 |
| *Aave_1238* | 0.34815 | homoserine dehydrogenase | 0.000670 |
| *Aave_2182* | -0.61854 | glyoxylate/hydroxypyruvate reductase A | 0.003011 |
| *Aave_2324* | 0.57658 | aspartate kinase | 3.92E-07 |
| *Aave_2433* | -0.71851 | dihydrolipoyl dehydrogenase | 2.95E-09 |
| *Aave_2464* | -0.38819 | dihydrolipoyl dehydrogenase | 7.00E-05 |
| *Aave_3649* | -0.33447 | phosphoglycerate dehydrogenase | 0.003302 |
| *Aave_4220* | -0.76233 | diaminobutyrate--2-oxoglutarate transaminase family protein | 2.30E-05 |
| *Aave_4221* | -0.58951 | aspartate aminotransferase family protein | 7.31E-07 |
| *Aave_4441* | 1.2117 | threonine dehydratase | 4.20E-16 |

Note: Differentially expressed genes (DEGs) related to amino acids metabolism, such as the pathways involved in valine, leucine and isoleucine degradation/biosynthesis, beta-Alanine metabolism, arginine and proline metabolism, alanine, aspartate and glutamate metabolism, glycine, serine and threonine metabolism in ΔyggS, compared to wild-type strain XjL12 were listed in the table. Gene Id: the locus tags of DEGs that identified by hits in a Blastn search against the *A. citrulli* AAC00-1 genome (NC_008752). FC: fold change


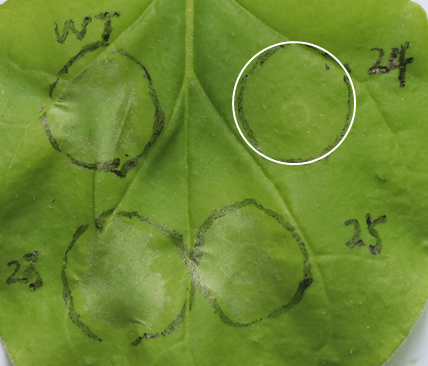


**Figure S1**. Screening transposon (Tn5)-insertion library by inoculating tobacco leaves. *A. citrulli* strains cultured in LB broth overnight were washed with sterilized water and were adjusted to OD600 of 0.3. Cell suspensions were injected into tobacco leaves by sterilized injection syringe. The image were acquired at 12 h post-inoculation (hpi). Among tested strains, a Tn5-insertion mutant ‘24’, later renamed as SK24, failed to elicit hypersensitive response (HR) at 12 hpi, while distinct HR were induced by WT at the mean time. White cycle indicated the region inoculated by SK24. WT: wild-type strain XjL12; 23-25: the Tn5-insertion mutants

**
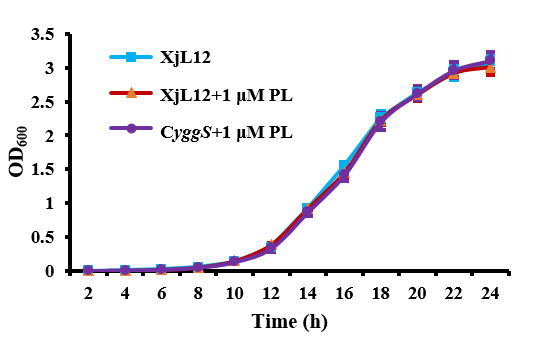
**

**Figure S2.** Effect of PL on XjL12 and C*yggS* growth. All strains suspensions were diluted to OD_600_=0.1 with sterilized water, and then transferred to fresh LB mixed with 1 μM PL at a ratio of 1:100. Optical density of cell suspensions was measured at 2 h intervals. The data points represent means and the tines represent the standard errors of the mean for three experiments. The experiment was repeated three times. XjL12: wild-type strain, C*yggS*: complemented strain of Δ*yggS*

**Sp**

**Gm**

**Cm**

**Amp**

**Tct**

**DCS**


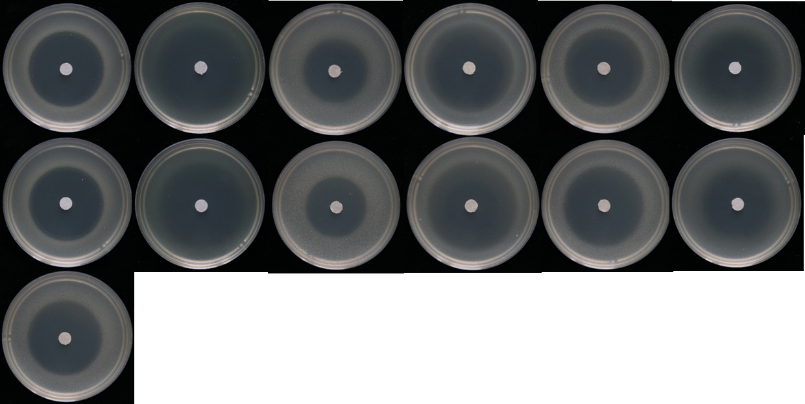


**XjL12**

**(pYggS)**

**Δ*yggS***

**XjL12**

**Figure** **S3**. Representative images illustrating the sensitivity of *A. citrulli* strains to the following antibiotics: Amp (ampicillin), Gm (gentamicin), Cm (chloramphenicol), Tct (tetracycline), Sp (spectinomycin), DCS (D-cycloserine). XjL12: wild-type strain of *A. citrulli*; Δ*yggS*: *yggS* deletion mutant; XjL12 (pYggS): *yggS*-overexpressing mutant.


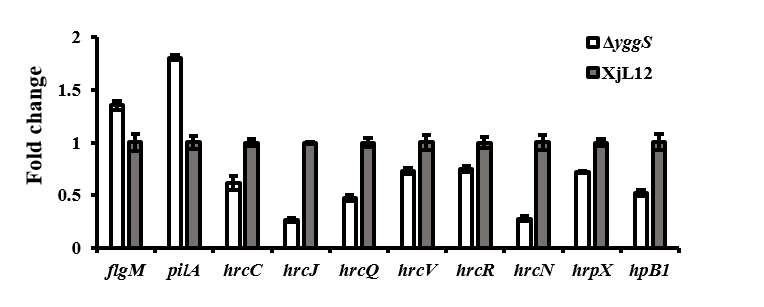


**Figure** **S4**. Expression level of selected genes between XjL12 and Δ*yggS*. To validate the accuracy of RNA-Seq data, ten genes related to motility and T3SS in the differentially expressed genes (DEG) list were selected for monitoring the expression level of those genes in mutant Δ*yggS* and wild-type strain XjL12 using qPCR. The corresponding locus tags in NCBI are as follows: *flgM*: *Aave_4418*; *pilA*: *Aave_4679*; *hrcC*: *Aave_0474*; *hrcJ*: *Aave_0466*; *hrcQ*: *Aave_0450*; *hrcV*: *Aave_0452*; *hrcR*: *Aave_0449*; *hrcN*: *Aave_0463*; *hrpB*: *Aave_0444*; *hrpB1*: *Aave_0479*.


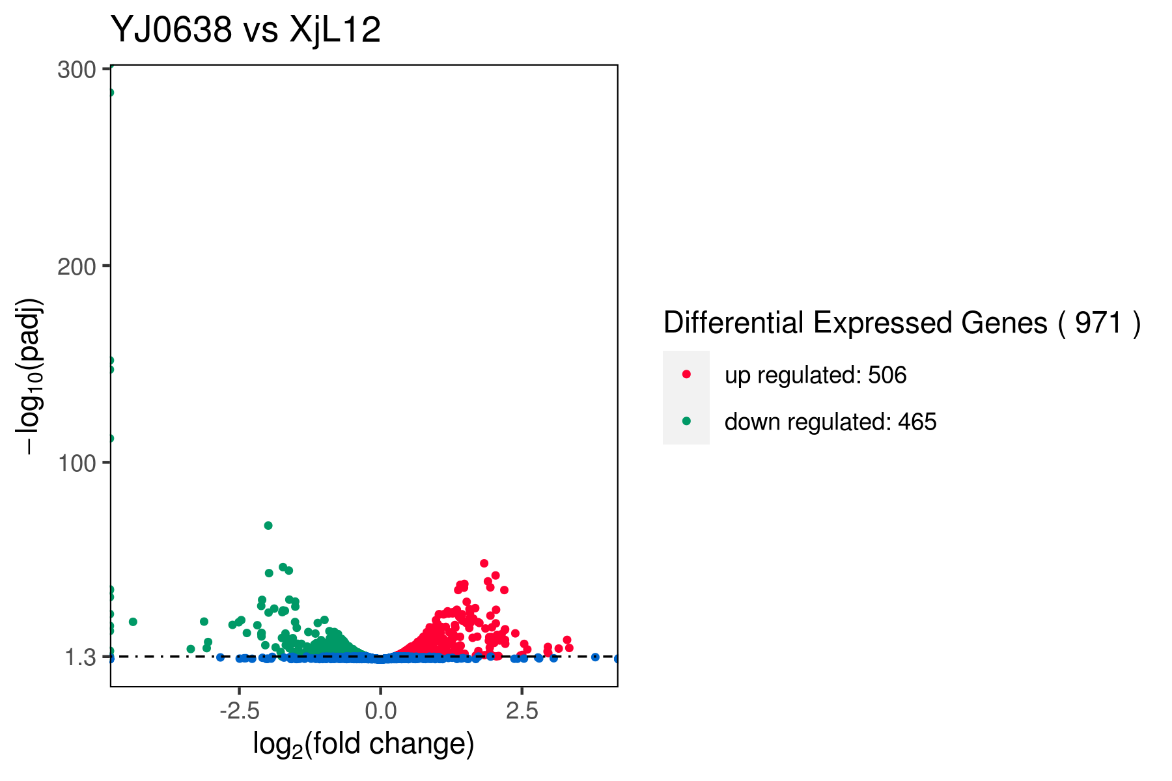


**Figure** **S5**. Volcano map of differential expressed genes (DEGs). Red, green and blue dot indicates up-regulated gene, down-regulated gene and gene expressed with no significance. The DEGs are screened in the condition of qvalue < 0.05. The vertical axis represents statistical significance of the difference of gene expression. The horizontal axis represents gene expression multiples in different samples.


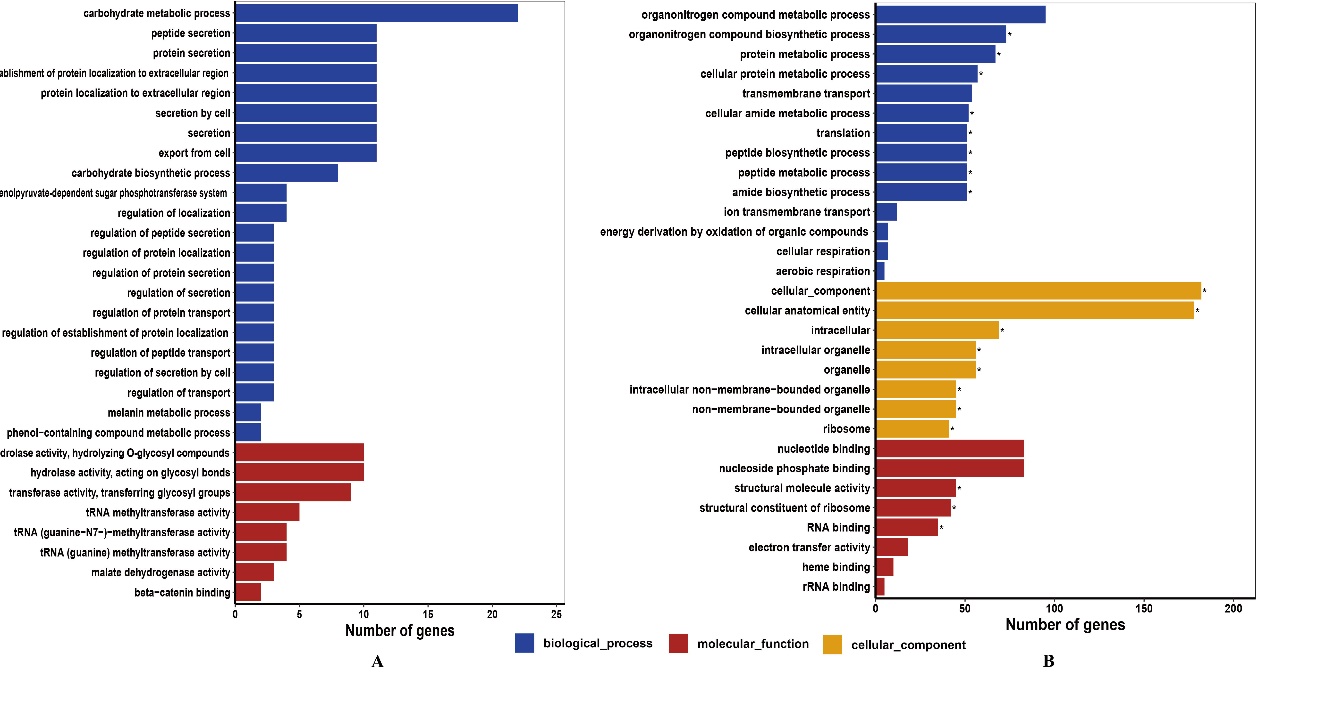


**A B**

**Figure** **S6**. Gene ontology (GO) of down-regulated differentially expressed genes (DEGs) **(A)** and up-regulated DEGs **(B)** between Δ*yggS* and wild-type strain XjL12. Only top 30 GO terms with most enrichment are shown in the image and divided into biological process, cellular component and molecular function, which is indicated by blue bar, yellow bar and red bar respectively. “ * ” indicates the significant enriched GO term. The vertical axis represents GO terms and the horizontal axis represents gene number.

**
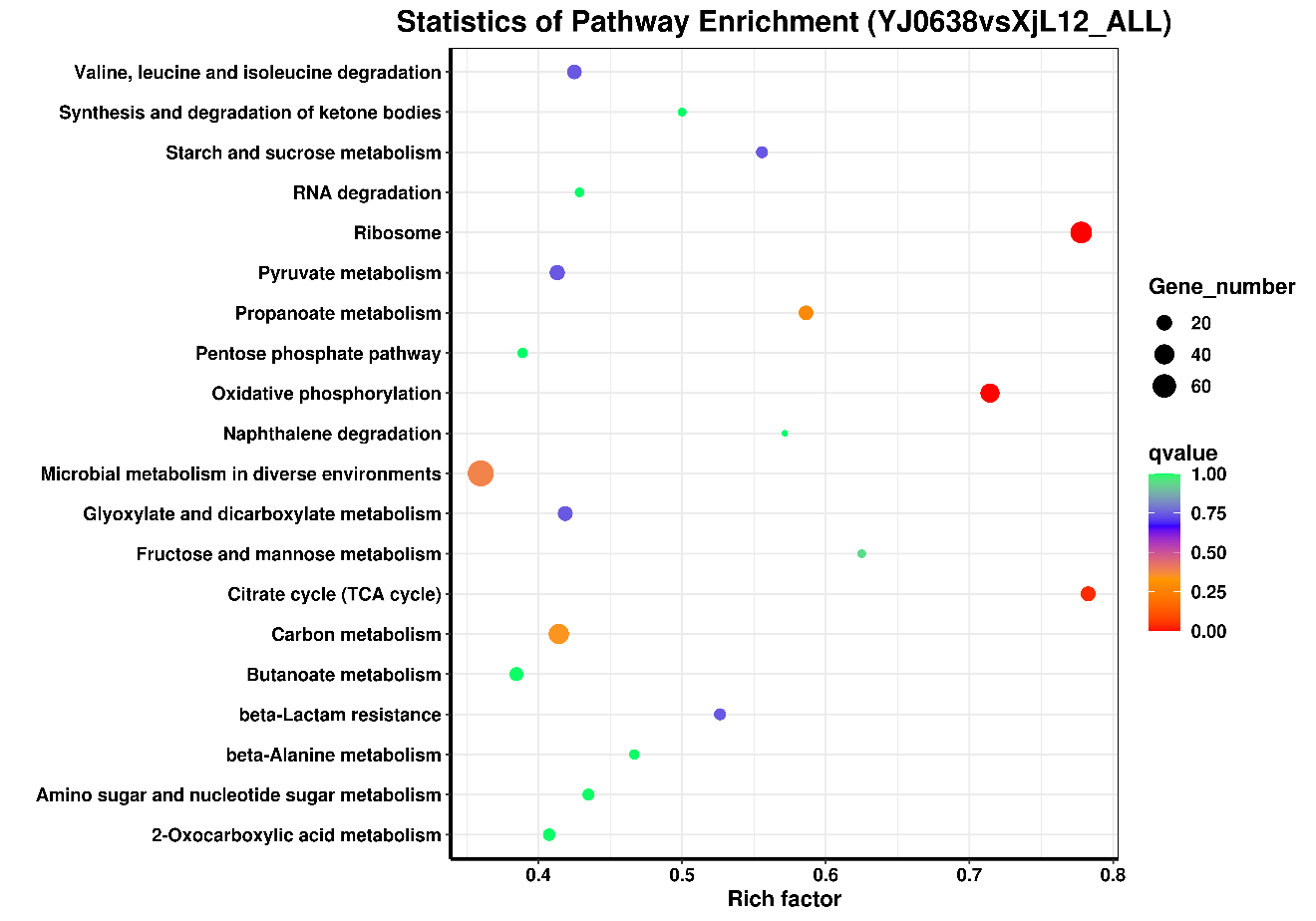
**

**Figure** **S7**. Statistics of Kyoto Encyclopedia of Genes and Genomes (KEGG) pathway enrichment. Only top 20 pathways with significant enrichment are shown in the image. Rich factor represents the ratio of the number of DEGs to the number of annotated genes in this pathway and the higher the rich factor is, the greater the enrichment degree is. The closer the qvalue is to 0, the more significant the enrichment is. The size of the dot indicates the number of differential genes in the pathway.

**Figure** **S8**. Expression level of flagella-related genes (*filA*, *filC*, *filS* and *filR*) between XjL12 amd XjL12 (pYggS). To determine the effect of overexpressed *yggS* on the expression of flagella-related genes, the expression levels of *filA*, *filC*, *filS* and *filR* were monitored in overexpression strain XjL12 (pYggS) and wild-type strain XjL12 using qPCR. The corresponding locus tags in NCBI are as follows: *filA*: *Aave_4416*; *filC*: *Aave_4400*; *filS*: *Aave_4398*; *filR*: *Aave_4383*.

**A**


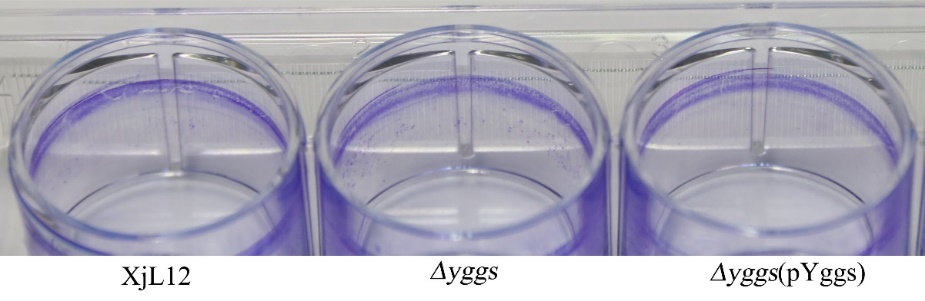


**C*yggS***

**XjL12**

**Δ*yggS***

**B**


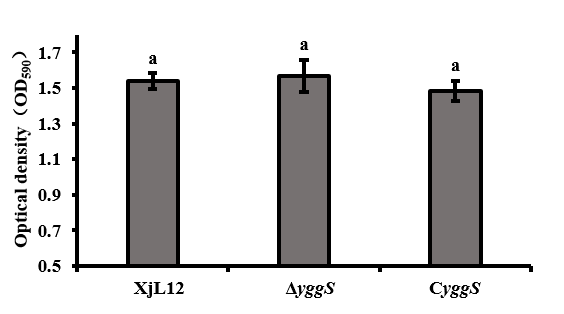


**Figure** **S9**. Effect of *yggS* on *Acidovorax citrulli* biofilm formation. **(A)** Images of biofilm formed in polystyrene culture plates characterized by stained methyl violet fringes around the well. **(B)** Biofilm quantification following washing of methyl violet stain with eluent (absolute ethyl alcohol) and optical density measurements at 590 nm. XjL12: the wild-type strain of *A.citrulli*, Δ*yggS*: ygg*S* deletion mutant of *A.citrulli*; C*yggS*: the complemented strain of Δ*yggS*. Letters above the bars represent significant differences as determined by the LSD test (*P* < 0.05).
