# Supplementary material for: yggS Encoding Pyridoxal 5′-Phosphate Binding Protein Is Required for Acidovorax citrulli Virulence
Source: Front Microbiol. 2022 Jan 11;12:783862. doi: 10.3389/fmicb.2021.783862 (PMC8787154; doi:10.3389/fmicb.2021.783862)
Supplement: Supplementary file 2 [file Data_Sheet_2.DOCX]

Plasmid rescue method was employed to identify the gene disrupted by Tn5. By backward sequencing analysis of the fragment containing Tn5 that was ligated with pUC19, the assembled nucleotide sequence was revealed and shown below. Letters in blue and in red indicate the nucleotides of transposon Tn5 and its flanking region, respectively. A pair of sequencing primers are shade with yellow. By Blastn online, the red nucleotides is the partly fragment of *Aave_0638*, encoding YggS family pyridoxal phosphate-dependent enzyme.

//…GGAACTCCTCGTACACCTTGCGCTGGGCGTCGCTCATGATGTCGTACACCATGGCGTGCACGGTCTTGTGGTCGAGGGCATCGACGTTGATGCGGCGCACGTCGCCATGCACGCGGATCATCGGAGGCAGGCCCGCGGACAGGTGCAGGTCGGAGGCCTTGTTCTTCACGCTGAAGGCGAGCAGTTGGGTGATGTCCACGAAAAACCCTTGGTCGTTTGGTACGCTTGAGAACAAACATTATGACGACGATTGGTAACAACCTCCAAGGGGTCCTGGACCGCATCGCGCGAGCCTGCGCACAGGCCGGCCGCGAGCCGTCCGGCGTGCGGCTGCTCGCCGTTTCCAAGACCTTCGGCGCCCCGTCCGTGCGCGAGGCGGCGCTGGCGGGGCAGCGGGCGTTCGGCGAGAACTACATCCAGGAGGGCGTCGAGAAGATCGCTGCCCTGGGCCTGTCTCTTGATCAGATCTGGCCGCCTAGGCCGCGGCCGCCGCGTTTAATGACCAGCACAGTCGTGATGGCAAGGTCAGAATAGCGCTGAGGTCTGCCTCGTGAAGAAGGTGTTGCTGACTCATACCAGGCCTGAATCGCCCCATCATCCAGCCAGAAAGTGAGGGAGCCACGGTTGATGAGAGCTTTGTTGTAGGTGGACCAGTTGGTGATTTTGAACTTTTGCTTTGCCACGGAACGGTCTGCGTTGTCGGGAAGATGCGTGATCTGATCCTTCAACTCAGCAAAAGTTCGATTTATTCAACAAAGCCACGTTGTGTCTCAAAATCTCTGATGTTACATTGCACAAGATAAAAATATATCATCATGAACAATAAAACTGTCTGCTTACATAAACAGTAATACAAGGGGTGTTATGAGCCATATTCAACGGGAAACGTCTTGCTCGAGGCCGCGATTAAATTCCAACATGGATGCTGATTTATATGGGTATAAATGGGCTCGCGATAATGTCGGGCAATCAGGTGCGACAATCTATCGATTGTATGGGAAGCCCGATGCGCCAGAGTTGTTTCTGAAACATGGCAAAGGTAGCGTTGCCAATGATGTTACAGATGAGATGGTCAGACTAAACTGGCTGACGGAATTTATGCCTCTTCCGACCATCAAGCATTTTATCCGTACTCCTGATGATGCATGGTTACTCACCACTGCGATCCCCGGGAAAACAGCATTCCAGGTATTAGAAGAATATCCTGATTCAGGTGAAAATATTGTTGATGCGCTGGCAGTGTTCCTGCGCCGGTTGCATTCGATTCCTGTTTGTAATTGTCCTTTTAACAGCGATCGCGTATTTCGTCTCGCTCAGGCGCAATCACGAATGAATAACGGTTTGGTTGATGCGAGTGATTTTGATGACGAGCGTAATGGCTGGCCTGTTGAACAAGTCTGGAAAGAAATGCATAAGCTTTTGCCATTCTCACCGGATTCAGTCGTCACTCATGGTGATTTCTCACTTGATAACCTTATTTTTGACGAGGGGAAATTAATAGGTTGTATTGATGTTGGACGAGTCGGAATCGCAGACCGATACCAGGATCTTGCCATCCTATGGAACTGCCTCGGTGAGTTTTCTCCTTCATTACAGAAACGGCTTTTTCAAAAATATGGTATTGATAATCCTGATATGAATAAATTGCAGTTTCATTTGATGCTCGATGAGTTTTTCTAATCAGAATTGGTTAATTGGTTGTAACACTGGCAGAGCATTACGCTGACTTGACGGGACGGCGGCTTTGTTGAATAAATCGAACTTTTGCTGAGTTGAAGGATCAGATCACGCATCTTCCCGACAACGCAGACCGTTCCGTGGCAAAGCAAAAGTTCAAAATCACCAACTGGTCCACCTACAACAAAGCTCTCATCAACCGTGGCTCCCTCACTTTCTGGCTGGATGATGGGGCGATTCAGGCCTGGTATGAGTCAGCAACACCTTCTTCACGAGGCAGACCTCAGCGCTATTCTGACCTTGCCATCACGACTGTGCTGGTCATTAAACGCGGCGGCCGCACTTGTGTATAAGAGTCAGGCCCTGGGCCAGCCGGAGTCCGGCCTCGCGCATCCGCTGGAGTGGCATTGCATCGGCCCCGTGCAGAGCAACAAGACCCGGCTGGTGGCGGAGCATTTCGACTGGGTGCACACGGTGGACCGCCTCAAGACGGCCGAGCGCCTGTCGCAGCAGCGGCCGGACCACCTGCCGGCACTGCAGCAGCTGCGAGTAATCGTCCAGGTCGCGCGCGACCACCACGAGCATGAAATCCTCGGGCCCGGAGATGCCGTGGAACATGACCACCTCGGGAATGGCGCACACCGCCTCCTCGAAAGGCAGCGACGAAGCTTCGGTCTGGTGGTCGATGCCCACCATCACGAACACCATCACTCCGAGTCCCAGCGCCCGGCGGTCGAGCGCGGCGTGGTAGCCCGCGATCACGCCTT…//
